# Structures of nonsense-mediated mRNA decay factors UPF3B and UPF3A in complex with UPF2 reveal molecular basis for competitive binding and for neurodevelopmental disorder-causing mutation

**DOI:** 10.1093/nar/gkac421

**Published:** 2022-05-31

**Authors:** Joshua C Bufton, Kyle T Powers, Jenn-Yeu A Szeto, Christine Toelzer, Imre Berger, Christiane Schaffitzel

**Affiliations:** School of Biochemistry, University of Bristol; University Walk, Bristol BS8 1TD, UK; School of Biochemistry, University of Bristol; University Walk, Bristol BS8 1TD, UK; School of Biochemistry, University of Bristol; University Walk, Bristol BS8 1TD, UK; School of Biochemistry, University of Bristol; University Walk, Bristol BS8 1TD, UK; School of Biochemistry, University of Bristol; University Walk, Bristol BS8 1TD, UK; Max Planck Bristol Centre for Minimal Biology, Cantock's Close, Bristol BS8 1TS, UK; School of Biochemistry, University of Bristol; University Walk, Bristol BS8 1TD, UK

## Abstract

UPF3 is a key nonsense-mediated mRNA decay (NMD) factor required for mRNA surveillance and eukaryotic gene expression regulation. UPF3 exists as two paralogs (A and B) which are differentially expressed depending on cell type and developmental stage and believed to regulate NMD activity based on cellular requirements. UPF3B mutations cause intellectual disability. The underlying molecular mechanisms remain elusive, as many of the mutations lie in the poorly characterized middle-domain of UPF3B. Here, we show that UPF3A and UPF3B share structural and functional homology to paraspeckle proteins comprising an RNA-recognition motif-like domain (RRM-L), a NONA/paraspeckle-like domain (NOPS-L), and extended α-helical domain. These domains are essential for RNA/ribosome-binding, RNA-induced oligomerization and UPF2 interaction. Structures of UPF2′s third middle-domain of eukaryotic initiation factor 4G (MIF4GIII) in complex with either UPF3B or UPF3A reveal unexpectedly intimate binding interfaces. UPF3B’s disease-causing mutation Y160D in the NOPS-L domain displaces Y160 from a hydrophobic cleft in UPF2 reducing the binding affinity ∼40-fold compared to wildtype. UPF3A, which is upregulated in patients with the UPF3B-Y160D mutation, binds UPF2 with ∼10-fold higher affinity than UPF3B reliant mainly on NOPS-L residues. Our characterization of RNA- and UPF2-binding by UPF3′s middle-domain elucidates its essential role in NMD.

## INTRODUCTION

**Figure 1.**
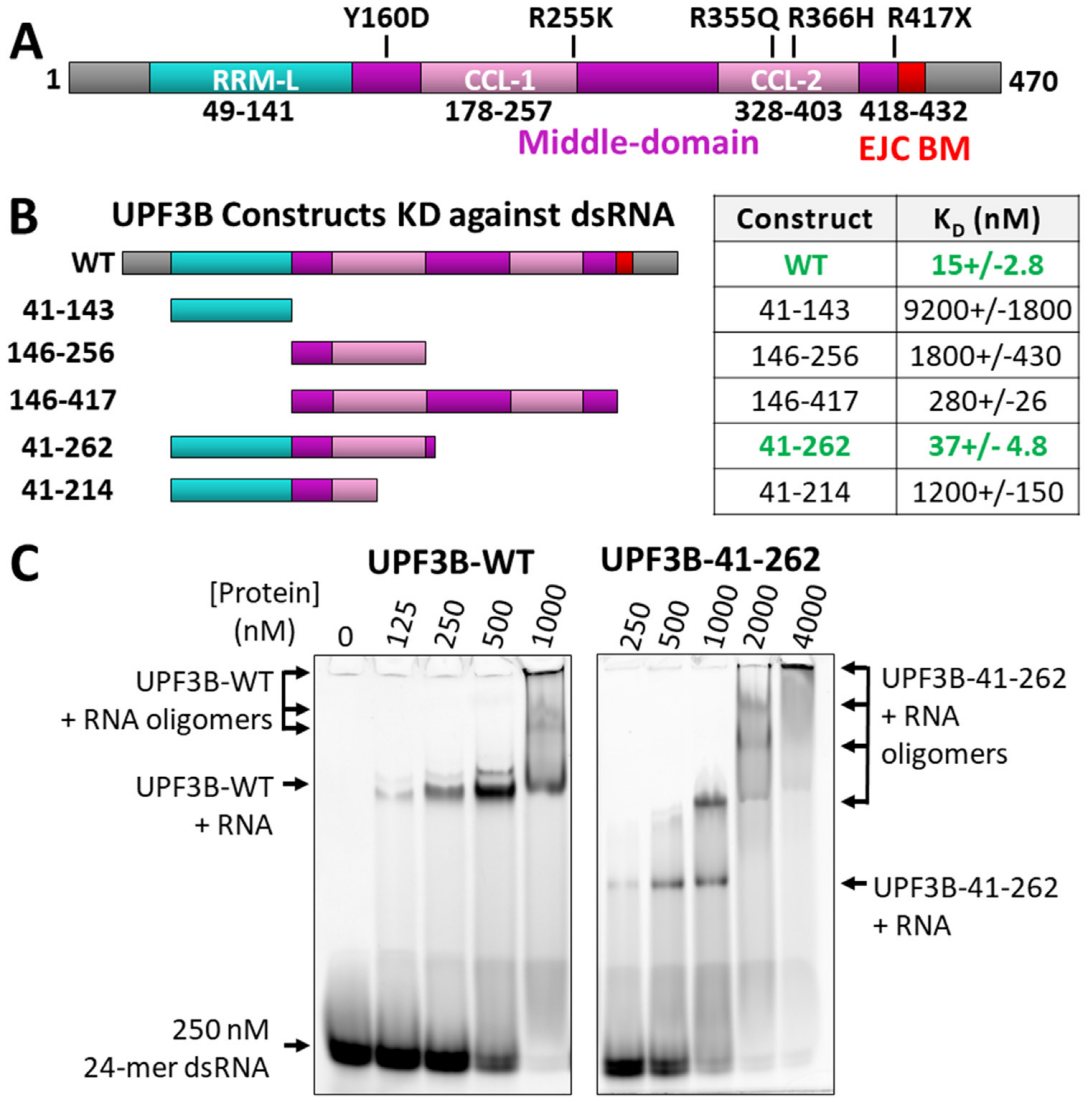
UPF3B domain architecture, RNA binding and RNA-induced oligomerization. (**A**) Schematic representation of UPF3B domain architecture predicted by homology modelling. (**B**) Schematic representation of the UPF3B constructs (left) utilized for testing dsRNA binding and corresponding dissociation constants determined by fluorescence anisotropy experiments (right) mapping the nucleotide-binding interface of UPF3B to its RRM-L and N-terminal part of the middle-domain (41-262) (binding curves in Supplementary Figure S7). (**C**) Electrophoretic mobility shift assays of UPF3B-WT (left) and UPF3B-41–262 (right) using double-stranded 24-mer RNA (dsRNA) indicating that UPF3B-41–262 retains RNA-induced oligomerization behaviour.

The nonsense-mediated mRNA decay (NMD) pathway targets mRNAs harbouring premature termination codons (PTCs) thus preventing translation of potentially toxic C-terminally truncated proteins (1–5). NMD is clinically highly relevant because nonsense mutations account for ∼20% of known disease-associated single base-pair substitutions (6). Simultaneously, NMD has a conserved and fundamental role in non-aberrant eukaryotic gene expression, for example in regulating neurodevelopment in mammals (7–9). The NMD machinery comprises the conserved UP-Frameshift proteins UPF1, UPF2 and UPF3 (paralogs A and B) (10–12). UPF3B is an auxiliary component of exon-junction complexes (EJC) that are deposited at exon-exon boundaries during mRNA splicing and act as enhancers of mammalian NMD (13,14). The interaction between the UPF proteins is critical for NMD activation. The UPF2–UPF3B complex binds to UPF1, leading to structural rearrangements and stimulation of ATPase and helicase activity of UPF1 (15,16). In addition, UPF2-UPF3B is suggested to activate SMG1 kinase-mediated UPF1 phosphorylation that triggers mRNA decay through recruitment of nucleases (17,18). More recently mRNA- and ribosome-binding of UPF3B has been reported (19,20). UPF3B was shown to slow down translation termination and support ribosome dissociation *in vitro* (19). Cross-linking studies revealed UPF3B-binding to mRNA upstream of exon-exon junctions (20) suggesting a role of UPF3B in the positioning of EJCs and the NMD machinery. However, due to the absence of structural and functional data, the precise mode of action of UPF3B in NMD remains elusive. UPF3B’s N-terminus comprises a conserved RNA recognition motif-like domain (RRM-L) lacking residues believed to be essential for high-affinity RNA interaction (21–23) (Figure 1A). Instead, the RRM-L binds the third middle-domain of eukaryotic initiation factor 4G **(**MIF4GIII) of UPF2 (12,21,24). The RRM-L domain is followed by a poorly characterized middle-domain and by a C-terminal EJC-binding motif (Figure 1A). The middle-domain has been implicated in binding release factor eRF3a and thus in UPF3B’s function in translation termination (19).

UPF3B missense mutations were identified in patients with autism, schizophrenia and X-linked intellectual disability (XLID) (25–29) (Figure 1A). Expression of these UPF3B mutants in neural stem cells impairs neuronal differentiation and reduces neurite branching (25). Point mutations Y160D and R366H, located in the middle-domain of UPF3B, result in subtly increased expression of NMD targets ARHGAP24 and ATF4 which are involved in down-regulation of neuronal branching and plasticity (25,30,31). UPF3B Y160 is highly conserved in vertebrates, *Drosophila melanogaster*, *Caenorhabditis elegans*, and also in vertebrate UPF3A, the paralog of UPF3B (25). The disease-causing UPF3B-Y160D mutation additionally leads to substantial upregulation of UPF3A. In many tissue types UPF3A is selectively cleared from cells but it can protect itself from degradation by interaction with UPF2 in the absence of functional UFP3B (28,32). The role of UPF3A in NMD remains enigmatic. UPF3A has been demonstrated as being capable of compensating for UPF3B loss-of-function on an NMD reporter substrate albeit with lower efficiency (33). In clinical cases, a greater stabilization of UPF3A in response to UPF3B dysfunction correlates with a less severe NMD dysfunction phenotype. This supports a view that UPF3A could compensate for UPF3B as an NMD activator (28). However, a loss-of-function and mouse knockout study in a wider transcriptome context showed decreasing nonsense mRNA stability in the absence of UPF3A, implicating UPF3A as an antagonist of NMD in mouse germ cells (34). Therefore, dissecting the functional interactions of the middle-domain of UPF3 and its contributions to the interplay between UPF3A and UPF3B is critically important to understand their role in NMD.

Here, we report two crystal structures of the RRM-L and N-terminal region of the middle-domain of UPF3B and UPF3A bound to the MIF4GIII domain of UPF2. Using these structures as a guide, we define the residues of both UPF3 paralogs involved in high-affinity interactions with UPF2. We show that the middle-domains of UPF3B and UPF3A adopt a NONA/paraspeckle-like (NOPS-L) fold followed by extended α-helical domains which are structurally homologous to paraspeckle proteins. In agreement with this homology, we observe RNA- and DNA-induced oligomerization of UPF3B as reported for paraspeckle proteins. We show that UPF3B’s binding of RNA and DNA is promiscuous, with preference for double-stranded RNA which is prominent in ribosomes. We map nucleic acid-binding and oligomerization activities onto the RRM-L and the N-terminal part of the middle-domain, encompassing the NOPS-L and first α-helical, coiled coil-like domain. The crystal structures of UPF3A and UPF3B domains in complex with UPF2-MIF4GIII reveal an unexpectedly intricate binding interface with UPF2-MIF4GIII wedged in between UPF3′s RRM-L and NOPS-L. The newly identified interactions between the middle-domain and UPF2 lead to a >200-fold affinity increase compared to UPF2-MIF4GIII and UPF3B’s RRM-L domain only. UPF3B-Y160 and adjacent conserved hydrophobic residues of the NOPS-L domain bind into a hydrophobic cleft of UPF2 previously implicated in binding nucleic acids (21). UPF3B-binding to UPF2 alters UPF2′s ability to form complexes with RNA and alters UPF3B-RNA complex stoichiometry. Notably, the UPF3B-Y160D mutation implicated in XLID reduces UPF3B’s affinity for UPF2-MIF4GIII ∼40-fold. UPF3A, which is upregulated in patients with the UPF3B-Y160D mutation, binds UPF2-MIF4GIII with even higher, picomolar affinity. This increase in affinity is mainly reliant on variations in UPF3A’s NOPS-L region as well as a subtle structural rearrangement in the RRM-L due to sequence differences. Interestingly, UPF3B Y160 can be phosphorylated in human cells highlighting a mechanism to finetune the interplay between UPF3B, UPF2 and UPF3A and to regulate NMD efficiency.

## MATERIALS AND METHODS

### Secondary structure prediction, homology modelling and sequence conservation analysis

Secondary structure predictions were generated using the primary amino acid sequence of UPF3B isoform 2, the predominant form found in cells, and submitted to Quick2D as well as PCOILS, MARCOIL and DeepCoil servers within the MPI Bioinformatics Toolkit (35). Highest scoring hits were considered those with a >95% probability of homology despite having 10–20% sequence identity with UPF3B including structures of proteins involved in splicing regulation: *Schizosaccharomyces pombe* Mei2, *Mus musculus* TIA-1, *Saccharomyces cerevisiae* PRP24, *Homo sapiens* RBM5, and ribosomal biogenesis factor *S. cerevisiae* NOP15 (PDB IDs: 6YYL, 2DGO, 2L9W, 2LKZ, and 3JCT, respectively). Homology modelling of UPF3B was conducted using HHPRED and MODELLER (36) servers using SFPQ PDB ID 4WIK to build the putative domains of UPF3B’s middle region. The WebLogo server was used to analyze sequence conservation across the middle-domain of UPF3B (37). The AlphaFold Protein Structure Database was used to obtain a prediction of UPF3B’s structure (38).

### Construct design and cloning

pFastBac-HTB (Invitrogen) baculoviral expression vectors for UPF3B-WT (residues 1–470), UPF3B-146–417 and UPF3B-146–256 were reported previously (19). UPF3B-41–143, UPF3B-41–189, UPF3B-41–214, UPF3B-41–262, UPF3A-58–206 (Iso1 and Iso2), UPF2-MIF4GIII (761–1054), and UPF2L (120–1227) were produced by PCR amplification (primers listed in Supplementary Table S1) using the Phusion High-Fidelity PCR kit (NEB #E0553S) prior to restriction digest and ligation into pPROEX-HTB (Invitrogen) vectors. UPF3B-41–189 derived point mutants including Y160A, Y160D, Y167A, Y167D, Y160A + Y167A, Y160D + Y167D and Avi-tagged Avi-UPF2-MIF4GIII as well as Avi-UPF3B-WT constructs were generated using a Q5 Site-Directed Mutagenesis Kit (NEB #E0554S) with mutagenic primers (Eurofins Genomics) (Supplementary Table S2). Fragments for UPF3A-58–206-Iso1 and UPF3A-58–173-Iso2 were codon-optimized for *Escherichia coli* expression and gene-synthesized (Twist Bioscience) prior to restriction digest and ligation into pPROEX-HTB (Invitrogen) vector.

### Protein expression and purification

UPF3B-WT, UPF3A-WT, Avi-UPF3B-WT, UPF3B-146–256 and UPF3B-146–417 were expressed using the MultiBac insect cell expression system (39). All other UPF3B, UPF3A, and UPF2 (MIF4GIII + UPF2L) constructs were transformed into *Escherichia coli* BL21 Rosetta2(DE3) (Novagen #71400) and grown in LB medium prior to induction with 0.3 mM isopropyl 1-thio-β-d galactopyranoside (IPTG) and expressed for 16 hours at 18°C with agitation. Cells were harvested by centrifugation at 1000 × g and 5000 × g for insect and bacterial cells, respectively, before flash freezing pellets for storage at –80°C.

For purification, cell pellets were thawed prior to resuspension in binding buffer (25 mM HEPES pH 7.4, 10 mM imidazole, 300 mM NaCl, 5% [v/v] glycerol) supplemented with 1× cOmplete EDTA free protease inhibitor cocktail tablet (Roche #11873580001). Cells were lysed via sonication, and the lysate clarified at 45 000 × g for 45 min at 4°C. The supernatant was applied onto a 5 ml HiFliQ Ni-NTA column (Generon #HiFliQ5-NiNTA-5). After washing the column with binding buffer, bound 6× His-proteins were eluted via a linear gradient from 10 to 350 mM imidazole (in binding buffer). Eluted protein-containing fractions were incubated with in-house purified Tobacco Etch Virus (TEV) protease for removal of the 6× His-tag. All proteins were further purified by reverse immobilized metal affinity chromatography to remove un-cleaved material prior to tandem ion exchange purification using a 5 ml HiTrap Q XL (Cytiva #17515901) column followed by a second cation exchange 5 ml HiTrap SP HP column (Cytiva #17115201). After loading the sample, the Q XL column was removed and elution carried out on the SP HP column using a linear gradient from 150 mM to 1 M NaCl in 25 mM HEPES pH 7.4, 2 mM β-mercaptoethanol. For UPF2-MIF4GIII and UPF2L constructs, ion exchange chromatography was carried out using the SP HP column only. Proteins were concentrated using Amicon centrifugal ultrafiltration units with a MWCO of 3 kDa. Protein concentrations were determined by measuring UV absorbance at 280 nm with calculated molecular weights and extinction coefficients using a NanoDrop One (ThermoFisher).

### CD spectroscopy assays

All CD measurements were performed in a 0.1 cm quartz cuvette using a J-1500 spectropolarimeter (Jasco) fitted with a Peltier temperature control unit. An initial CD wavelength scan measurement at 25°C was carried out with 10 μM of UPF3B-146–256 and UPF3B-146–417 dialyzed in CD buffer (100 mM KCl, 10 mM potassium phosphate pH 7.4). CD spectra were acquired across a wavelength range of 195–260 nm collecting data at 0.1 nm intervals. For the temperature wavelength scan, CD measurements at 222 nm were recorded at 0.2°C intervals from 5 to 75°C with heating at 5°C/min and a 10 second equilibration time at each temperature point. A buffer-only derived baseline was subtracted from all datasets.

### Fluorescence anisotropy assays

Hexachlorofluorescein-labelled (HEX) DNA and RNA oligonucleotide probes (Supplementary Table S1) were chosen based on previous work (19) (Eurofins Genomics) and diluted to 5 nM in assay buffer (150 mM NaCl, 25 mM HEPES pH 7.45, 1 mM TCEP). An assay volume of 150 μl was dispensed into a Hellma 10 × 2 mm Suprasil quartz cuvette (Merck #Z802778). For double-stranded substrates, complementary oligonucleotides were mixed at a 1:1 molar ratio and heated to 95°C prior to slowly cooling to room temperature overnight in an insulated chamber. Proteins were then titrated into the cuvette at increasing concentrations and fluorescence anisotropy measurements were recorded at 20°C with a Jobin Yvon Fluorolog (Horiba Scientific) with excitation and emission wavelengths of 530 and 550 nm, respectively. Measurements were taken with an integration time of 0.5 s and averaged across four accumulations. Dilutions and titrations were carried out in triplicate and averages with standard deviations were plotted before fitting the data (GraphPad Prism) with a single-component binding equation ${\rm{Y\;}} = ( {{\rm{Bmax*}}\frac{{\rm{X}}}{{{\rm{KD}} + {\rm{X}}}}} ){\rm{\;}} + {\rm{C\;}}$ to determine the dissociation binding constant (*K*_D_) where *B*_max_ is the maximum anisotropy change (saturated anisotropy value – starting value), *X* is the concentration of protein, and *C* is the anisotropy value at which the X axis value is 0.

### Electrophoretic mobility shift assays (EMSAs)

For UPF3B-nucleic acid interaction analysis, HEX-labelled oligos (Supplementary Table S1, Eurofins Genomics) were diluted to 250 nM in EMSA buffer (25 mM HEPES pH 7.45, 2 mM MgCl_2_, 150 mM NaCl, 0.5 mM TCEP). A high (250 nM) probe concentration was required to detect band shifts due to multiple oligomeric species. 1:1 serial dilutions of UPF3B-WT were then added to the nucleotide solutions and incubated on ice for 30 mins before supplementing with 7.5% (final) glycerol and running on a Novex 6% Tris–glycine WedgeWell gel (Invitrogen #XP0006A) equilibrated in Novex Tris-Glycine Native Running Buffer at 4°C. For UPF3B–UPF2–ssRNA interactions, HEX-labelled ssRNA (Supplementary Table S1) was diluted to 250 nM in EMSA buffer along with either 2 μM UPF3B-WT or UPF2L and increasing ratios of either UPF2L or UPF3B. Incubations were performed on ice for 1 h before loading on a 4–20% Novex WedgeWell Tris-Glycine native gel prior to Coomassie blue staining. Gels were imaged using a G:Box F3 gel doc system (Syngene) for Coomassie staining and Typhoon FLA 9500 (GE Healthcare) imager with an excitation wavelength of 532 nm using a BPG1 emission filter for HEX-labelled oligo detection.

### Surface plasmon resonance assays (SPR)

Experiments were performed on a Biacore T200 using a SA-Series S sensor chip (Cytiva #BR100531). Avi-UPF2-MIF4GIII and Avi-UPF3B-WT were biotinylated via incubation with BirA as previously described (40) prior to size exclusion chromatography using a Superdex S75 Increase 10/300 GL column (Cytiva # 29148721) to remove free biotin. Immobilization of biotinylated ligands was carried out on a single flow cell leaving a second flow cell as a background control for signal subtraction. All analysis was performed at 15°C. For UPF2–UPF3 interaction analysis, 350 RU of biotinylated-Avi-UPF2-MIF4GIII was immobilized. UPF3B and UPF3A construct serial dilutions in Biacore running buffer (300 mM NaCl, 25 mM HEPES pH 7.4, 0.25 mM TCEP, 0.05% Tween-20) were injected at 30 μl/min with an association phase of 180 s and a dissociation phase of 240 s. After every dissociation, the surface was regenerated with a 120 s injection of regeneration solution (1 M MgCl_2_, 25 mM HEPES pH 7.4, 0.05% Tween-20, 5% glycerol). Resulting sensorgrams were analyzed with the Biacore evaluation software (version 1.0) yielding on- and off-rates obtained through a global fit of both the association and dissociation phases of at least four different concentrations of each analyte using the 1:1 binding model.

For testing ribosome subunit binding, 150 RU of biotinylated Avi-UPF3B-WT was immobilized. A dilution series of 40S / 60S subunits was prepared in ribosomal suspension buffer (200 mM KCl, 2.5 mM Mg(OAc)_2_, 20 mM HEPES pH 7.6, 0.5 mM TCEP, 10 mM NH_4_Cl, 0.05% Tween-20) and injected at 20 μl/min for 6 min followed by a 12 min dissociation phase. The surface was regenerated by two 90 sec injections of regeneration solution (1 M KCl, 20 mM HEPES pH 7.4, 0.05% Tween-20, 20 mM EDTA) prior to the next injection. Data was analyzed by steady state fit in the Biacore evaluation software by measuring the RU values of each concentration 20 sec before the end of the association phase. Equilibrium dissociation constants (*K*_D_) were determined by plotting the RU values as a function of analyte concentration and fitted with a single-component binding equation in GraphPad Prism ${\rm{\;Y\;}} = ( {{\rm{Bmax*}}\frac{{\rm{X}}}{{{\rm{KD}} + {\rm{X}}}}} ){\rm{\;}} + {\rm{C}}$. Reported *K*_D_ values are an average of three or more experimental repeats with ranges reported by standard deviation.

### Protein crystallization

We performed crystallization experiments of various UPF3B truncations alone, with RNA oligomers, and with UPF2-MIF4GIII. The latter was mixed at a 1:1 molar ratio with UPF3B truncation constructs encompassing RRM-L and parts of the middle-domain (UPF3B-41–189, UPF3B-41–214, and UPF3B-41–262) prior to purification via size-exclusion chromatography (SEC) using a Superdex 200 10/300 GL column (Cytiva #17517501) equilibrated with GF buffer (25 mM HEPES pH 7.4, 300 mM NaCl, 2 mM TCEP). Crystallization experiments were carried out using the standard sitting drop vapor diffusion method on LOW PROFILE Swissci Polystyrene Triple Drop Plates (Molecular Dimensions # MD11-003LP-100) containing 45 μl of reservoir solution. Using a mosquito crystal (SPT Labtech), 125, 150 and 175 nl of UPF3B-41.189 + UPF2-MIF4GIII at an equimolar complex concentration of 15 mg/ml in GF buffer was dispensed along with 175, 150 and 125 nl of reservoir solution in drops A, B and C respectively resulting in 300 nl total drop volume. Plates were incubated at both 4°C and 20°C. Crystallization of UPF3A-58–208 followed the same protocol as that used for UPF3B-41–189. We had several crystallization hits for the UPF3B-41–189 and UPF3A-58–206 constructs with UPF2-MIF4GIII in the initial screens. The best diffracting crystals appeared after 7 days at 20°C in 0.1 M calcium acetate, 0.1 M MES pH 6.0, 15% w/v PEG 400 for UPF3B-UPF2 and after 10 days at 20°C in 0.1 M HEPES Sodium pH 7.0, 10% w/v PEG 4000 and 10% v/v 2-Propanol for UPF3A-UPF2. Both continued to grow over the course of a week prior to harvesting. Drops were supplemented with 20% Ethylene Glycol (final concentration) upon harvesting as a cryoprotectant prior to flash freezing in liquid nitrogen.

### Data collection and structure determination

Diffraction data were collected on the IO4-1 (UPF3B) and I24 (UPF3A) beamlines at the Diamond Light Source under nitrogen cryo-stream (∼100 K) (Harwell Science and Innovation Campus) and images processed using XDS (Version: 01/2020) and scaled with AIMLESS (41,42). The crystals diffracted to a resolution of 2.6 Å in space group *P*4_1_22 for UPF3B and 2.95 Å in space group *P*12_1_1 for UPF3A, both with four complexes in the asymmetric unit. The structure of UPF3B-41–189 + UPF2-MIF4GIII was phased by molecular replacement in PHASER using the previously solved UPF3B-RRM + UPF2-MIF4GIII structure (PDB ID:1UW4) as an input model (21), while UPF3A was phased using the UPF3B-41–189 + UPF2-MIF4GIII model. The four copies of UPF3B-UPF2-MIF4GIII heterodimers in the asymmetric unit were found to be very similar having an average RMSD of 0.3 Å and 0.4 Å respectively between complexes. Manual model building was performed in Coot (version 0.8.9) and automated refinement carried out in PHENIX (version 1.17.1-3660) (43,44). Models were validated and statistics obtained using MolProbity (45). Figures were prepared using PyMol (version 2.3.4).

## RESULTS

### UPF3B’s domain architecture is homologous to paraspeckle proteins

To infer potential structure and function of the hitherto uncharacterized middle-domain of UPF3B (Figure 1A), we computationally analyzed this domain. Quick2D (35) provided a strong consensus indicating several interspersed α-helical regions in the middle-domain (Supplementary Figure S1). Strikingly, two of these stretches (CCL-1 and CCL-2) are predicted to extend over 70 and 60 residues (P178-P257 containing the disease-causing mutation R255K mutation and S328-K390 comprising the R355Q and R366H mutations). DeepCoil and MARCOIL servers (46,47) also indicated that these regions have a >90% probability to form coiled coil like structures (not shown).

To identify related proteins of known structure within the Protein Data Bank, we submitted the full-length UPF3B sequence to HHpred (48). Two of the highest scoring hits were for crystal-derived structures of the human splicing factor proline- and glutamine-rich protein (SFPQ) (49) aligning to UPF3B’s RRM-L domain and extending to residue 250 in the middle-domain (Supplementary Figure S2A) (50). SFPQ is a member of the Drosophila Behaviour/Human Splicing (DBHS/ paraspeckle) protein family implicated in subnuclear body formation, transcription, and splicing (51). The DBHS family is structurally characterized by two tandem N-terminal RRM domains, followed by a NonA/paraspeckle domain (NOPS) and a C-terminal coiled-coil (Supplementary Figure S2B,C) (52). The latter, along with the NOPS and RRM domains, facilitates homo- and hetero-dimerization (Supplementary Figure S2B) (53). In contrast, size exclusion chromatography-multiangle laser light scattering–refractometry (SEC-MALLS) analysis indicates that UPF3B is a monomer in solution (19). HHpred aligns UPF3B’s RRM-L domain to the second RRM domain of SFPQ (Supplementary Figure S2C) and residues 145–250 of UPF3B’s middle-domain onto the NOPS and coiled-coil regions of SFPQ (Supplementary Figure S2A–C). We generated a SFPQ-based homology model of UPF3B using MODELLER (36) which comprised the RRM-L domain followed by a NOPS-like linker connecting to an α-helical stretch with negative and hydrophobic residues that could facilitate self-association with the RRM-L (Supplementary Figure S3A). The α-helical region extends into a solvent-exposed, uninterrupted stretch of mostly positively charged residues spanning the length of the modelled helix (Supplementary Figure S3A). A model generated by AlphaFold (38) also predicted the existence of these α-helices, spanning nearly identical regions (P178-I253 as well E338-K406) and sharing similar electrostatic surface potential characteristics (Supplementary Figure S3B,C). However, it should be noted that these helical predictions are not within regions of very high confidence (defined as per-residue confidence score > 90%), and thus may be considered unreliable and may not exist in the absence of a binding partner.

We produced two UPF3B middle-domain variants expanding over different lengths of the predicted α-helical regions (residues 146–256 and 146–417). Circular dichroism (CD) spectra presented a canonical double dip at 210 and 222 nm characteristic for α-helical structure (54) (Supplementary Figure S3D), with a larger shift in CD signal observed for the longer construct. Temperature wavelength scans showed that these constructs unfolded in an unusual non-sigmoidal fashion, indicating gradual unravelling rather than globular domain unfolding, in agreement with the middle-domain adopting an extended α-helical structure (Supplementary Figure S3D). The relative proportion of secondary structure from the obtained CD spectra for residues 146–417 (UPF3B’s middle domain) was analyzed by BeStSel (55) indicating that ∼42% consists of α-helices, ∼36% is disordered or loops, and the remainder is potentially β-strand. This is in good agreement with our structural predictions supporting long α-helices and substantial disorder in the middle-domain.

### UPF3B middle-domain binds preferentially to double-stranded RNA and displays RNA-induced oligomerization

Members of the paraspeckle family have been described as ‘functional aggregators’ with the ability to form large oligomers in the presence of DNA (49,52). They act as molecular scaffolds for a large range of different nucleic acids and protein binding partners (49,52). Therefore, we tested UPF3B’s interactions with RNA and DNA oligomers in electro-mobility shift assays (EMSAs) starting with a 24-mer hairpin-RNA oligonucleotide that previously co-eluted with UPF3B in size exclusion chromatography experiments (19). Like paraspeckle proteins (49), UPF3B displayed multiple nucleic acid-binding events leading to the formation of large oligomers unable to migrate into the gel at increased UPF3B concentrations (Supplementary Figure S4A). Affinities for different nucleic acid substrates to UPF3B were determined using fluorescence anisotropy (FA)-based binding assays. The resulting curves show that UPF3B has a ∼six-fold higher affinity for single-stranded RNA (ssRNA, 30 nM) compared to ssDNA (190 nM) (Supplementary Figure S4B, C). Intriguingly, UPF3B bound double-stranded (ds) oligomers with considerably higher affinities than their single-stranded counterparts, with dsRNA and dsDNA displaying two-fold (15 nM) and ∼3-fold (61 nM) higher affinities respectively (Supplementary Figure S4D, E). With dsRNA being UPF3B’s preferred substrate, we next explored binding to human 40S and 60S ribosomal subunits using surface plasmon resonance (SPR) as these are rich in dsRNA, including double-stranded RNA extension segments (Supplementary Figure S5). In agreement with previously reported UPF3B-binding to ribosomes (19,20), we observed high-affinity interactions with both 40S (38 nM) and 60S (4.3 nM) ribosomal subunits.

### Nucleic acid binding is mediated by UPF3B’s RRM-L and middle-domain

To determine UPF3B’s minimal RNA-binding domains, stable middle-domain fragments (Figure 1B, Supplementary Figure S6) with and without the RRM-L domain were tested in FA-assays (Figure 1B, Supplementary Figure S7) using a dsRNA oligomer (Supplementary Table S1). The RRM-L domain alone (UPF3B-41–143) displayed poor affinity for dsRNA (*K*_D_ of 9.2 μM) in FA-assays, corroborating previous EMSA data that found no interaction between UPF3Bs RRM-L and RNA under their experimental conditions (21). This can be attributed to UPF3B’s lack of typical RNP2 motif residues that are implicated in canonical RRM–RNA interactions (21). This weak affinity for RNA in tandem with demonstrated affinity for UPF2 supports this RRM-L as being primarily suited for protein-protein rather than protein-nucleic acid interactions. A construct spanning the predicted NOPS-L region and the first coiled-coil-like region of the middle-domain (UPF3B-146–256) revealed only modestly higher affinity (1.8 μM) when compared with the RRM-L alone. An extension of this fragment to the complete middle-domain containing both predicted coiled-coil like regions (UPF3B-146–417) bound dsRNA with 280 nM affinity. A construct including the RRM-L, NOPS-L and the first coiled-coil-like region (UPF3B-41–262) was sufficient to bind dsRNA with close to wildtype affinity (37 nM versus 15 nM for UPF3B-WT) (Figure 1B). Consistently, UPF3B-41–262 retained wildtype-like RNA-induced oligomerization evidenced by multiple band shifts in EMSA (Figure 1C). Further truncation of the middle-domain (UPF3B-41–214) by removal of 48 residues of the first coiled-coil-like region (Supplementary Figure S3C, black box) resulted in >30-fold decreased affinity (*K*_D_ of 1.2 μM, Figure 1B). This region (residues 214–262) comprises 24 arginines and lysines, which all could contribute to high-affinity RNA binding (Supplementary Figure S3C). Taken together, our experiments suggest that RRM-L, NOPS-L and the first coiled-coil-like region are essential for UPF3B’s ability to bind dsRNA.

### Structure of UPF3B in complex with UPF2-MIF4GIII

**Figure 2.**
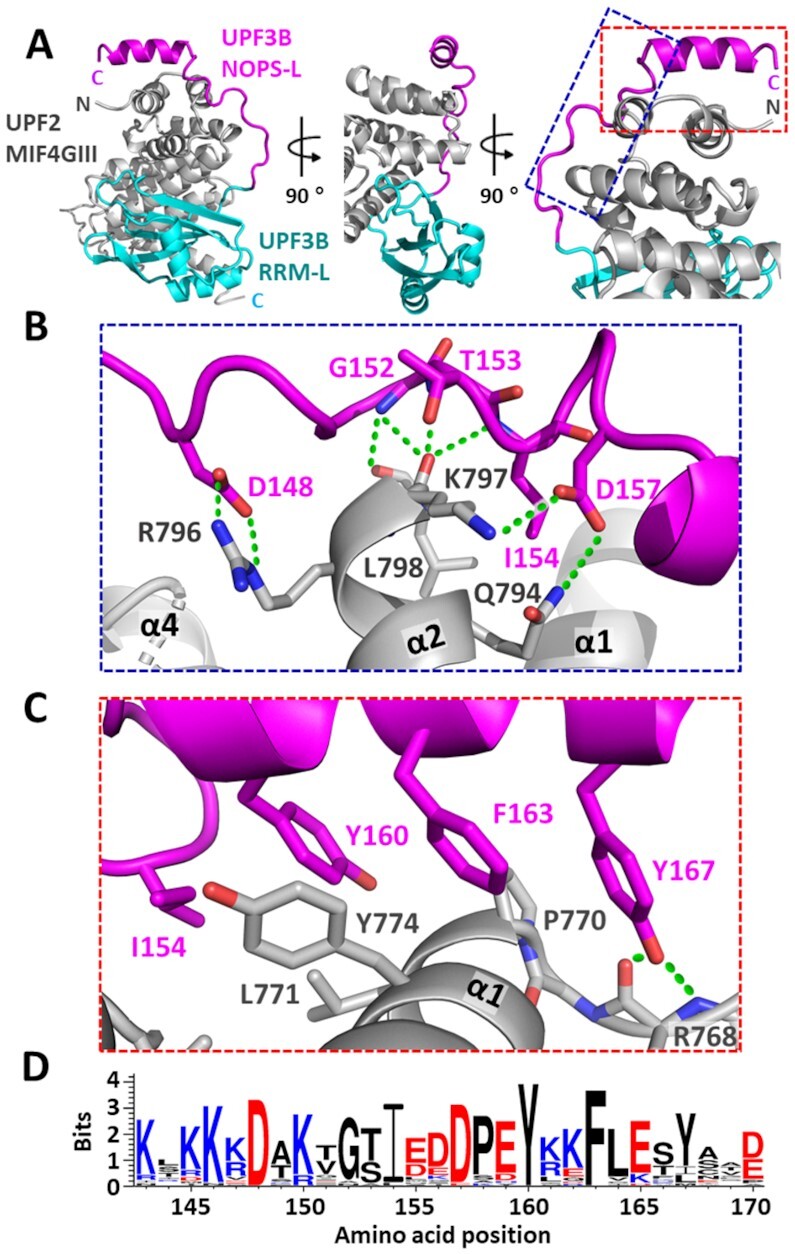
Crystal structure of UPF3B-41–189 + UPF2-MIF4GIII complex. (**A**) Cartoon representation of the crystallographic model with three representative views of UPF2-MIF4GIII (grey) complexed with UPF3B-41–189 comprising the RRM-L domain (cyan), the NOPS-L linker and α-helix (magenta). (**B**) Zoomed view (blue box, panel A) showing the residues involved in interactions between the NOPS-L linker with UPF2. (**C**) Zoomed view (red box, panel A) of residues in the NOPS-L α-helix contributing to binding of UPF2. Polar and ionic interactions are indicated (green lines). (**D**) WebLogo (37) alignment summarizing sequence conservation across a range of UPF3B homologs (Supplementary Figure S9) for the NOPS-L region indicating conservation of several of the residues involved in complex formation. The accumulative height of each stack (in bits) indicates the degree of conservation at that position. The height of each individual letter indicates the frequency that amino acid is found at that position.

To study the architecture of UPF3B’s middle-domain we crystallized a construct comprising UPF3B residues 41–189, comprising the RRM-L and the N-terminal part of the middle-domain (NOPS-L), in complex with UPF2-MIF4GIII and solved the structure to 2.6 Å resolution (Table 1). In good agreement with the predictions from AlphaFold and homology modelling (Supplementary Figure S3A,B), the beginning of the middle-domain of UPF3B adopts a NOPS-L domain consisting of a linker followed by an α-helix (Figure 2A).

**Table 1.** Refinement statistics of UPF3B-41–189 + UPF2-MIF4GIII and UPF3A-58–206 + UPF2-MIF4GIII crystal structures

**Data collection**		
Complex	UPF3B-41–189 + UPF2-MIF4GIII	UPF3A-58–206 + UPF2-MIF4GIII
PDB code	7NWU	7QG6
Wavelength (Å)	0.9795	0.9999
Synchrotron (beamline)	I04 Diamond Light Source	I24 Diamond Light Source
**Cell dimensions**		
Space group	*P*4_1_22	P12_1_1
*a, b, c (*Å)	130.59, 130.59, 267.33	77.68, 108.58, 119.94
α, β, γ (°)	90.00, 90.00, 90.00	90.00, 90.10, 90.00
**Collection statistics**		
Resolution range (Å)	2.6–65.30	2.95–66.70
Completeness (%)	99.84	99.20
*R*_merge_	0.041 (0.443)	0.072 (0.473)
*R*_meas_	0.0575 (0.627)	0.102 (0.669)
CC 1/2	0.999 (0.720)	0.993 (0.580)
Signal-to-noise-ratio (I/σI)	11.77 (1.60)	7.31 (2.12)
Total reflections	1 173 009	138 788
Unique reflections	80 565	41 711
Multiplicity	14.4	3.3
Refinement statistics		
*R*_work_ (%)	21.67	20.69
*R*_free_ (%)	25.59	26.33
RMS bonds (Å)	0.004	0.003
RMS angles (°)	0.536	0.470
Ramachandran favoured	96.54	93.92
Ramachandran allowed	3.46	6.08
Ramachandran outliers	0.00	0.00
Rotamer outliers	0.00	1.61
Wilson *B*-factor (Å^2^)	60.92	66.91
Clash score	4.22	1.52
MolProbity score	1.43	1.46
**Number of non-hydrogen atoms**		
Total	12 248	11 757
Protein/water/ligands/ions	12 043/160/45/0	11 707/19/30/1

**Figure 3.**
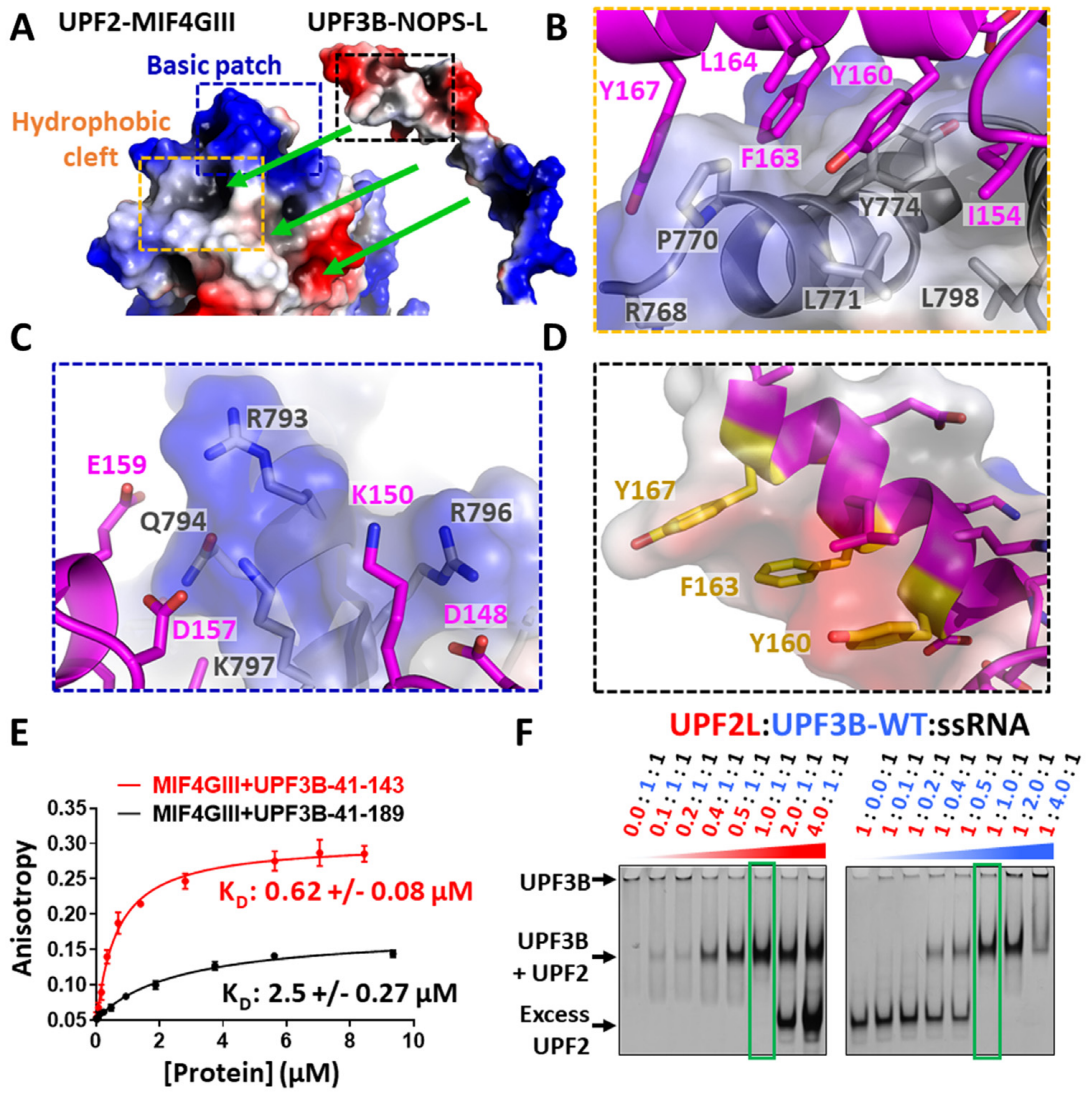
UPF3B-UPF2 interface analysis. (**A**) Electrostatic surface potential of spatially separated chains of UPF2-MIF4GIII and the NOPS-L domain of UPF3B derived from our crystal structure. Blue, red and white surface indicates positive, negative, and hydrophobic surface potential, respectively. Green arrows indicate how NOPS-L binds to UPF2-MIF4GIII regions. (**B**) Zoom in of hydrophobic cleft (orange box, panel A) interactions between UPF3B-NOPS-L (magenta cartoon) and UPF2-MIF4GIII (electrostatic surface and grey cartoon). (**C**) Zoom in of UPF2-MIF4GIII basic patch residues (blue box, panel A) and their interactions with UPF3B-NOPS-L. (**D**) Zoom in of UPF3B-NOPS-L α-helix residues (black box, panel A) involved in hydrophobic interaction with UPF2-MIF4GIII hydrophobic cleft (including Y160). Aromatic residues selected for mutagenesis are highlighted in yellow. (**E**) Fluorescence anisotropy binding curves of the complex of the RRM-L domain (UPF3B-41–143) with UPF2-MIF4GIII (red curve) and the complex crystallized in this study (UPF3B-41–189 + UPF2-MIF4GIII) (black curve) to a HEX-labelled 24mer ssRNA solution, indicating an inhibition of UPF2-RNA binding in the presence of UPF3B-41–189 relative to UPF3B-41–143. Protein titrations were carried out in triplicate and error bars plotted via standard deviation before fitting a single component binding equation in GraphPad Prism to calculate *K*_D_ values. (**F**) Coomassie-stained native 4–20% Novex gels loaded with ssRNA + UPF3B-WT incubated with increasing amounts of UPF2L (left) and with ssRNA + UPF2L incubated with increasing amounts of UPF3B-WT (right). Green boxes highlight UPF3B:UPF2 complexes at 1:1 stoichiometry.

The area of the structure spanning the RRM-L domain and its interactions with UPF2 align very well with the previously reported UPF3B RRM-L–UPF2-MIF4GIII structure (21) displaying an RMSD of 0.44 Å. In agreement, only minor changes are observed in UPF3B and UPF2-MIF4GIII (Supplementary Figure S8), and the same residues are involved in protein-protein interaction (21). The following NOPS-L linker region is composed of conserved residues D148, G152, T153, I154 and D157 which form multiple polar and salt-bridge interactions with R796, K797, L798, and Q794 of helices α1 and α2 of UPF2-MIF4GIII (Figure 2B). The subsequent α-helix in the NOPS-L further contributes to UPF2 interaction, primarily through hydrophobic contacts with P770, L771 and Y774 in helices α1 and α2, involving UPF3B residues I154, Y160 and F163. Y167 additionally forms polar contacts with R768 of UPF2-MIF4GIII at the end of the resolvable portion of the helix (Figure 2C, Figure 3A, B). Alignments of UPF3B and UPF3A show that this region (residues 143–170) is highly conserved in plants and animals (56,57), with particularly strong conservation for residues D148, I154, D157, Y160, F163 and Y167 which are all involved in UPF2 interaction (Figure 2D, Supplementary Figure S9). In summary, the NOPS-L region contributes to binding of UPF2-MIF4GIII via highly conserved UPF3B residues.

### UPF3B interferes with RNA binding by UPF2-MIF4GIII

Conserved acidic residues D148, D157 and E159 of UPF3B are all interacting with or in close proximity of a basic patch of UPF2-MIF4GIII comprising residues R793, R796, K797 and Q794 (Figure 3A, C). This basic patch of UPF2 was previously suggested to bind RNA *in vitro* (21). Considering that these residues coordinate UPF3B in our structure, we postulated that UPF2-MIF4GIII RNA binding would be impaired when in complex with UPF3B-41–189. In agreement, we observed a 4-fold decrease in UPF2-MIF4GIII affinity for ssRNA (2.5 μM) in the presence of UPF3B-41–189 compared to UPF3B RRM-L alone (0.62 μM) or in the absence of UPF3B (0.7 μM) in FA assays (Figure 3E). We conclude that the UPF3B’s NOPS-L domain outcompetes ssRNA for UPF2-MIF4GIII binding.

### UPF2 interferes with RNA-induced oligomerization of UPF3B

We next investigated UPF2–UPF3B complex formation in the presence of RNA via EMSAs using a larger UPF2 construct with wildtype activity (UPF2L, amino acids 120–1227) (15,16). UPF3B-WT was incubated with ssRNA concentrations that lead to formation of large oligomers in EMSAs (Figure 3F, Supplementary Figure S4A, left). Subsequent addition of equimolar amounts of UPF2L dissolved the large oligomers and led to formation of UPF3B–UPF2L complexes with defined stoichiometry (Figure 3F, green box). *Vice versa*, titration of UPF3B-WT into a fixed concentration of ssRNA and UPF2L leads to formation of defined UPF2L–UPF3B complexes and at higher concentrations to formation of larger oligomers which sequester UPF2L (Figure 3F). Together, this data suggests that UPF2 alters the RNA-binding behaviour of UPF3B and inhibits the formation of UPF3B–RNA oligomers.

### UPF3B-Y160 is critical for high-affinity UPF2 binding

**Figure 4.**
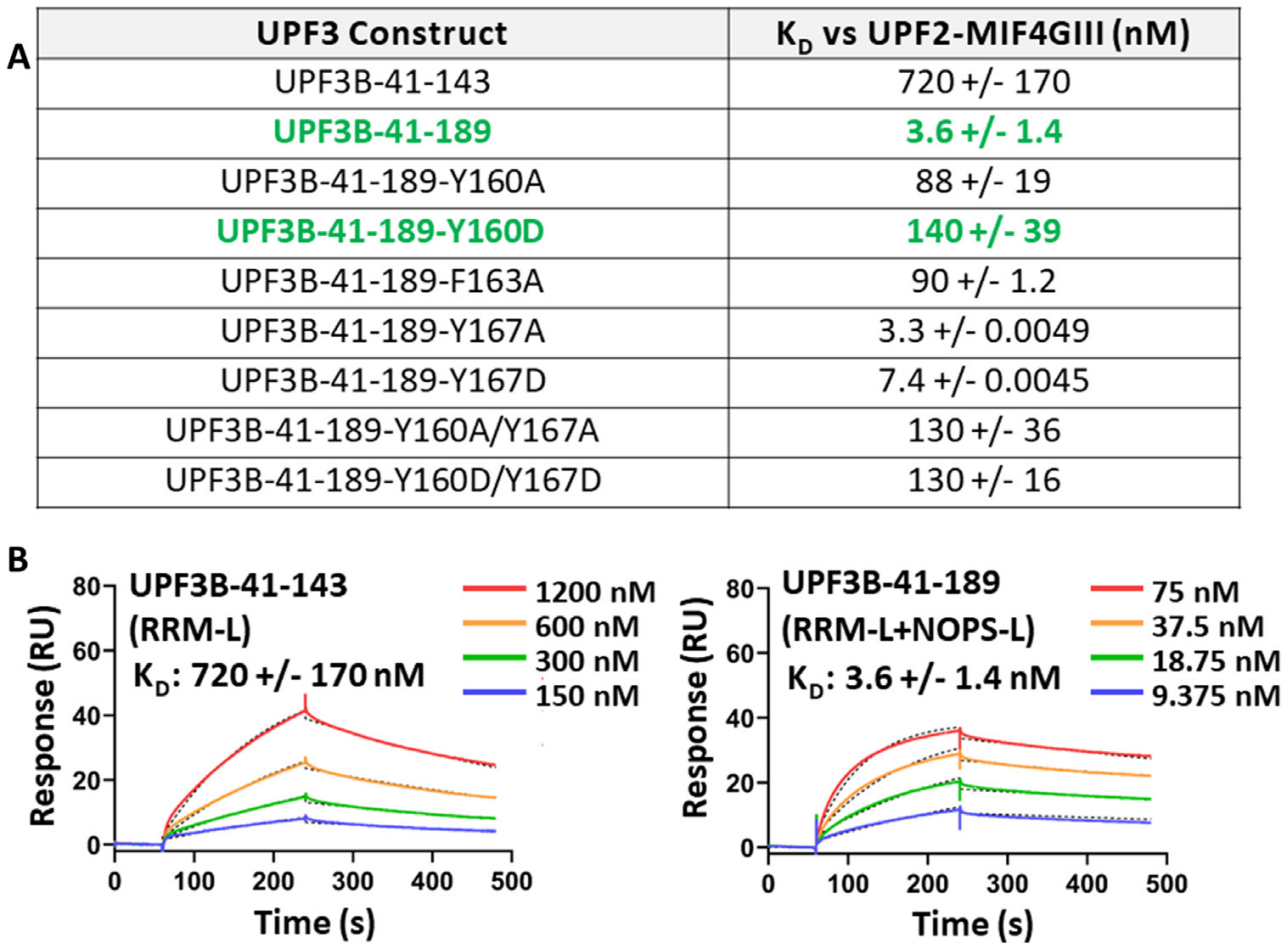
Characterization of interaction of UPF3B variants with UPF2-MIF4GIII. (**A**) Table summarizing the *K*_D_ values determined in this study by SPR between immobilized UPF2-MIF4GIII and UPF3B constructs, highlighting a ∼200-fold increase in affinity of UPF3B-41–189 relative to the RRM-L alone as well as a ∼40-fold affinity decrease due to Y160D mutation (green). (**B**) Representative binding curves of UPF3B-41–143 (RRM-L only) and UPF3B-41–189 are shown. Sensorgrams for additional mutation analyses are shown in Supplementary Figure S11.

We further explored the contribution of the NOPS-L domain to the UPF3B–UPF2 interface. Using SPR, we determined a *K*_D_ between UPF3B-41–189 and UPF2-MIF4GIII of 3.6 nM, which is 200-fold stronger than the *K*_D_ for the RRM-L alone (720 nM) (Figure 4A,B). The contribution of the conserved residues Y160, F163 and Y167, which are buried in a hydrophobic cleft formed by UPF2-MIF4GIII residues P770, L771, Y774, L798 (Figure 3A, B, D) was investigated next. These residues are important because UPF3B Y160D is implicated in XLID disease phenotypes (25,26,28), and UPF3B residues Y160 and Y167 can be phosphorylated in human cells (58,59) and also in our recombinant UPF3B expressed in insect cells (Supplementary Figure S10).

We tested UPF2-MIF4GIII binding of UPF3B-41–189 mutants Y160A, Y160D (phosphomimetic/disease mutation), F163A, Y167A, Y167D (phosphomimetic), and double mutants Y160A + Y167A and Y160D + Y167D (Figure 4A, Supplementary Figure S11). UPF3B’s Y160A and Y160D mutants reduced the affinity for UPF2-MIF4GIII to 88 nM (24-fold reduction) and to 140 nM (39-fold reduction) respectively compared to 3.6 nM for UPF3B-1–189. Similarly, the F163A mutation led to a *K*_D_ of 90 nM (25-fold reduction). In contrast, UPF3B mutations Y167A and Y167D had close to no impact, resulting in *K*_D_’s of 3.3 and 7.4 nM for UPF2-MIF4GIII respectively (Figure 4A). Consistently, UPF3B double mutants behaved like single Y160 mutants with both Y160A/Y167A and Y160D/Y167D having *K*_D_’s of ∼130 nM (Figure 4A). In summary, phosphorylation of UPF3B’s tyrosine residue Y160 or mutation to aspartate results in ∼40-fold reduced UPF2-binding affinity, likely by preventing accommodation of this residue into the hydrophobic cleft formed by UPF2-MIF4GIII.

### Impact of UPF3A isoforms on UPF2 binding

**Figure 5.**
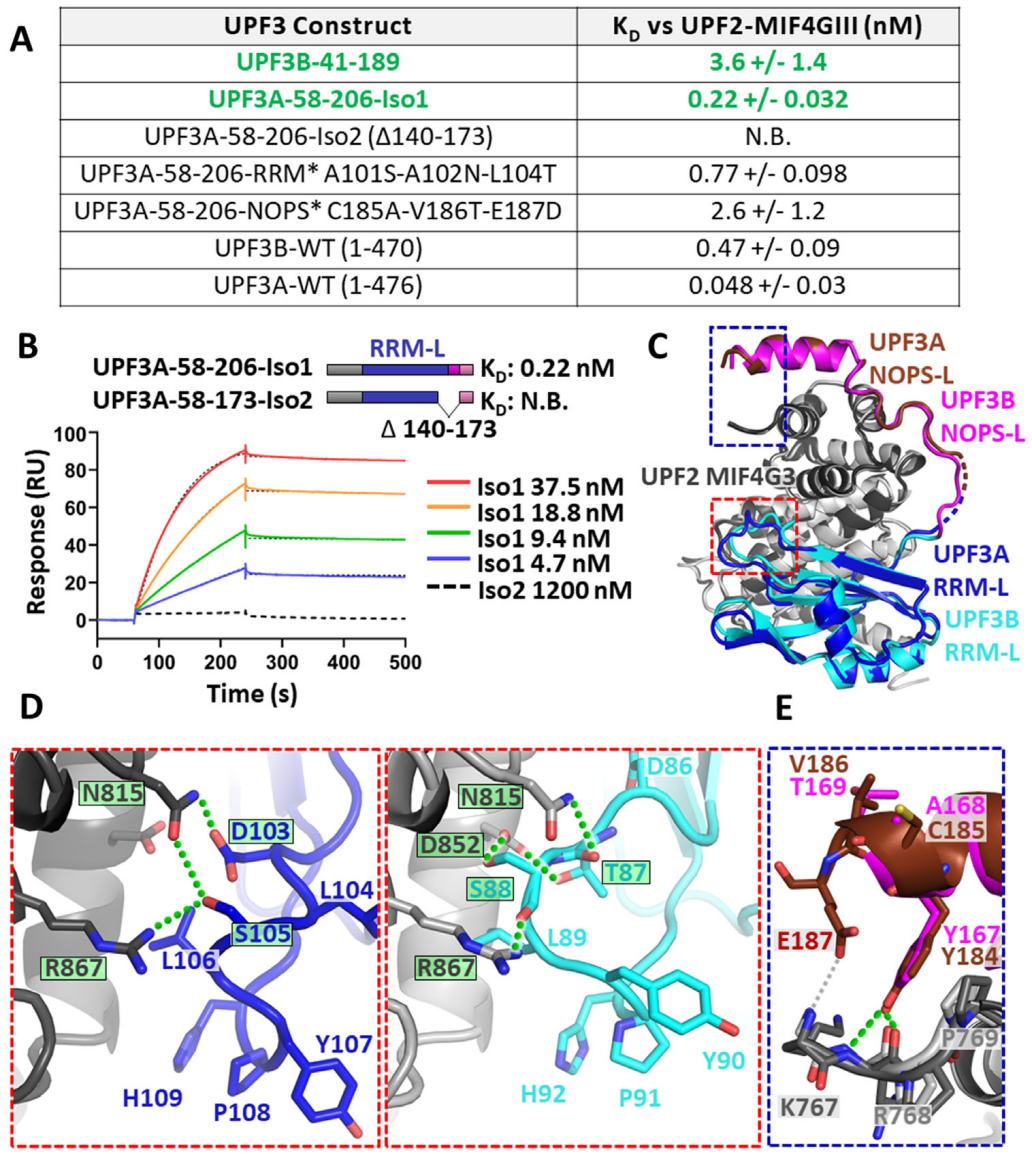
Characterization of UPF3A interaction with UPF2-MIF4GIII. (**A**) Table summarizing the *K*_D_ values between immobilized UPF2-MIF4GIII and UPF3B and UPF3A constructs, as determined by SPR. The higher affinity of UPF3A-58–206 compared to UPF3B-41–189 is highlighted (green). N.B. stands for no binding. (**B**) Representative binding curves of UPF3A (isoform variants 1 and 2) are shown. SPR of additional constructs is shown in Supplementary Figure S12. (**C**) Cartoon representation of the crystallographic model UPF2-MIF4GIII (grey) complexed with UPF3A-58–206 comprising the RRM-L domain (dark blue), the NOPS-L linker and α-helix (dark red) overlaid with UPF3B-41–189 comprising the RRM-L domain (cyan) and the NOPS-L region (magenta). (**D**) Zoomed view (red box, panel C) of residues in the RRM-L which undergo a structural rearrangement in the case of UPF3A (left panel) relative to UPF3B (right panel). Polar and ionic interactions are indicated (green lines) and corresponding residues are boxed and highlighted in green. (**E**) Zoomed view (blue box, panel C) highlighting the potential interaction (grey line) of E187 of UPF3A with K767 of UPF2 (dark red).

UPF3B Y160D mutation leads to an upregulation of UPF3A protein levels in affected families (25,28,32). Two splice variants of UPF3A exist in humans; isoform 1 which retains exon4 and isoform 2 which excludes exon4 (Δ141–173) thereby deleting the β5 strand of the RRM-L and the entire NOPS-L domain (Figure 5A,B). We generated corresponding UPF3A constructs with the same boundaries as UPF3B-41–189 (UPF3A-58–206-Iso1, UPF3A-58–173-Iso2). Surprisingly, UPF3A-58–206-Iso1 had an affinity of 220 pM for UPF2-MIF4GIII, binding 16-fold tighter than UPF3B-41–189 (Figure 5A, B). To corroborate this finding, we next used full-length UPF3A-WT and UPF3B-WT in SPR analyses (Supplementary Figure S12) and determined affinities of 470 pM for UPF3B-WT and 48 pM for UPF3A-WT for UPF2-MIF4GIII, indicating a similar higher affinity (∼10-fold) for UPF3A (Figure 5A).

In contrast, UPF3A-58–173-Iso2 showed no measurable response in SPR assays (Figure 5A,B), explainable by the deletion of a large part of the UPF2-interaction surface. In agreement, previous studies find that unlike UPF3A-isoform 1, isoform 2 is inactive in NMD and unable to bind UPF2 in co-immunoprecipitation experiments (33,60).

### Structure of UPF3A in complex with UPF2-MIF4GIII

To further analyze UPF3A’s higher affinity to UPF2-MIF4GIII, we crystallized UPF3A-58–206-Iso1 with UPF2-MIF4GIII (Figure 5C). Resulting crystals diffracted to a resolution of 2.95 Å and were phased using the UPF3B-41–189 + UPF2-MIF4GIII structure. As expected, the architecture of UPF3A and UPF3B is highly conserved as well as the interface between UPF3A and UPF2-MIF4GIII (Figure 5C). A previous structure (21) described the contact surface between the RRM-L of UPF3B and the MIF4GIII of UPF2 as being reliant on K52, R56, R57, E132, Q137, K138 and F136 of UPF3B forming salt bridges and polar interaction with D847, R854, E851 and E858. This contact interface is unchanged in both our UPF3B and UPF3A structures (all residues involved are conserved) offering no explanation of the discrepancy of affinities between the two isoforms (Supplementary Figure S13). However, deviations between the two crystal structures are found at the binding interfaces where sequence conservation between UPF3B and UPF3A diverges.

One such region is a 6-residue hairpin of the RRM-L between β2 and β3 of UPF3B and UPF3A (N85 and T87 changed to A102 and L104 in UPF3A) where a shift in the register occurs (Figure 5C,D). In UPF3B, the interacting surface is mainly polar, with UPF2 residues N815, D852 and R867 interacting with the backbone of T87, the side chains of T87 and S88, and the backbone of S88, respectively. UPF3B’s L89 binds to a hydrophobic pocket comprised of UPF2 residues L855, V859 and F864. In UPF3A, the register shift spanning residues 102–109 (85–92 in UPF3B) (Figure 5D, compare left and right panels) alters the positions of side chains of both UPF2 and UPF3A. The side chain of residue D103 (D86 of UPF3B which is not involved in binding) binds the pocket and forms a polar contact with the side chain of N815. Moreover, S105 (S88 of UPF3B) forms a polar contact with R867 and an additional polar contact with the side chain of N815. L106 (L89 in UPF3B) still resides within the same hydrophobic pocket formed by UPF2. Taken together, this orientation may support a moderately stronger interaction reliant on the additional polar contact between D103 with N815, which is not evident in the UPF3B structure.

A further region with small structural alterations between UPF3B-UPF2 and UPF3A-UPF2 is the NOPS-L linker region (Figure 5C). It forms a virtually similar network of polar and ionic interactions in both complexes despite the low sequence conservation of this UPF3 region. In the NOPS-L α-helical region of the UPF3A structure, the terminal end is better resolved relative to the UPF3B structure. This enabled extended modelling of the NOPS-L-UPF2 interface, albeit with some side-chains being better resolved in some chains relative to others depending on crystal packing context. The residues of UPF3B A168, T169 and D170 are poorly resolved and/or entirely absent in UPF3B, while this equivalent region of UPF3A, residues C185, V186, and E187 respectively, is better resolved (Figure 5E). UPF3A E187 could form an additional polar interaction with the backbone of UPF2′s K767, likely stabilizing the terminal end of the NOPS-L binding interface. This polar interaction would be absent in UPF3B due to the shorter side chain of D170 (no discernible density in our UPF3B structure). This potential additional interaction could result in a more extended and likely more stable binding interface between UPF3A and UPF2.

### UPF3A NOPS-L and RRM-L domain variations lead to stronger UPF2 interaction relative to UPF3B

To explore the relative contributions of the structural alterations in the two complexes and to identify the basis of UPF3A’s ∼10-fold higher affinity to UPF2, we performed structure-guided mutagenesis and SPR experiments. We produced two UPF3A constructs which mimic UPF3B via localized mutations in the RRM-L β-hairpin (UPF3A-58–206-RRM* A101S-A102N-L104T) and the NOPS-L α-helical region (UPF3A-58–206-NOPS* C185A-V186T-E187D). In SPR experiments, the RRM* construct indicated a 3.5-fold reduction in affinity to UPF2-MIF4GIII (K_D_ of 0.77 nM) compared to UPF3A wildtype. The NOPS* construct had a *K*_D_ of 2.6 nM corresponding to a ∼12-fold reduction in UPF2 binding. This affinity is very close to the 3.6 nM affinity of UPF3B-41–189 to UPF2-MIF4GIII. Therefore, the aspartate to glutamate mutation in the NOPS-L region of UPF3A contributes to the higher affinity of UPF3A for UPF2 given the limited divergence between their sequences and structural organization. In summary, UPF3A binds with ∼10-fold higher affinity than UPF3B, mainly relying on an additional interaction between the NOPS-L and UPF2-MIF4GIII domains, and to a lesser extent on a structural rearrangement in the β2-β3 hairpin of the RRM-L domains.

## DISCUSSION

Here, we characterize UPF3′s middle-domain and reveal that UPF3 shares structural and functional homology with paraspeckle/DBHS proteins comprising an RRM-like domain, a NOPS-like linker and extended α-helical regions. Like paraspeckle proteins, UPF3B binds DNA and RNA and shows oligomerization induced by nucleic acid-binding. Importantly, UPF2 binding involves the same paraspeckle-like regions. The crystal structure of UPF3B with UPF2-MIF4GIII reveals that the NOPS-L region forms additional contacts to UPF2 required for high-affinity interaction between UPF2 and UPF3B. Thus, the N-terminal portion of the middle-domain (i) contributes to a dramatic (200-fold) increase in affinity for UPF2 (Figure 4), (ii) modulates UPF2′s ability to interact with nucleic acids by binding to a hydrophobic groove in MIF4GIII suggested to bind RNA (Figure 3), and (iii) is required for RNA-binding and RNA-induced oligomerization of UPF3B (Figure 1). We determined a corresponding crystal structure of UPF3B’s paralog UPF3A with UPF2-MIF4GIII demonstrating that the domain architecture is conserved in UPF3 and that UPF2 is bound involving the same domains.

**Figure 6.**
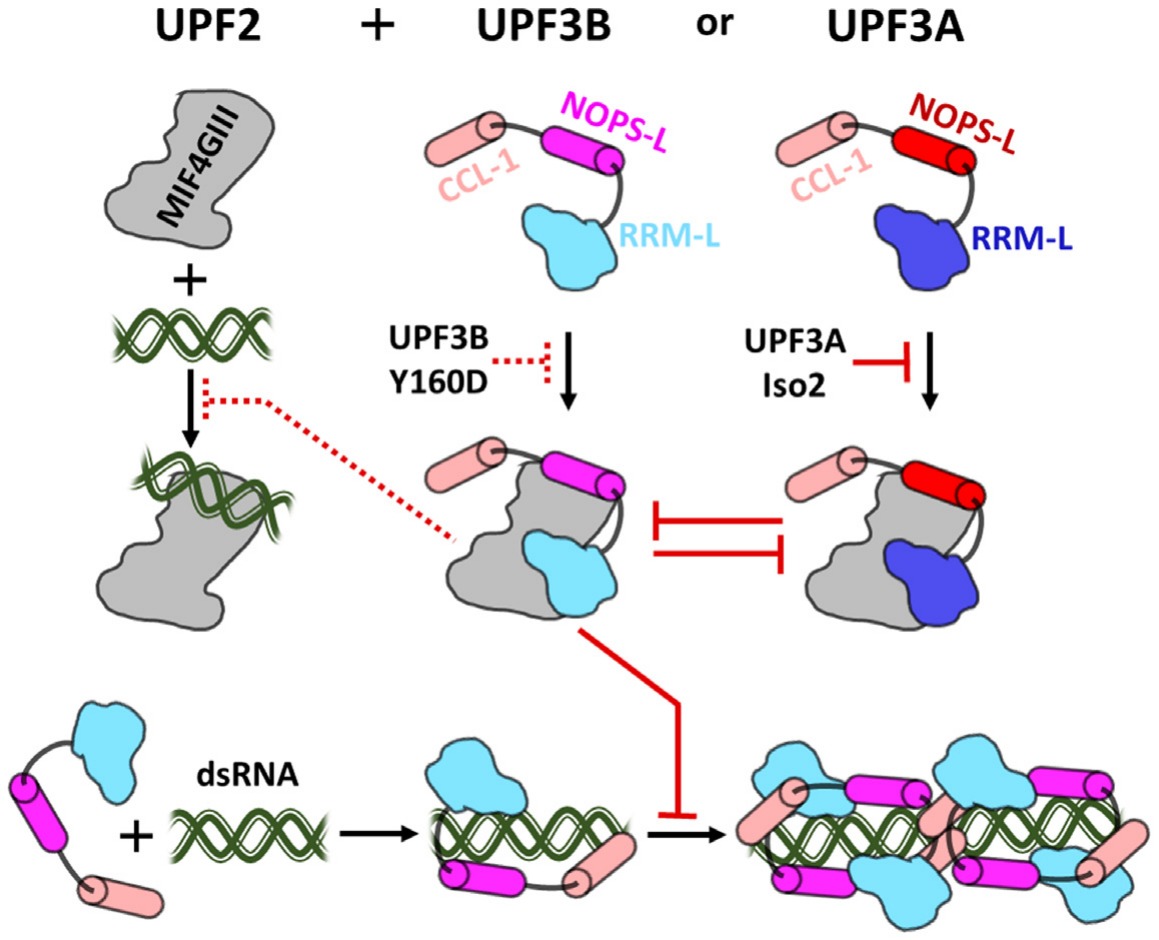
UPF3′s interactions with UPF2 and RNA. UPF2′s MIF4GIII domain (grey) can interact with dsRNA (green), with UPF3B (cyan and magenta) or UPF3A (blue and red). Formation of UPF2-UPF3 complexes interferes with RNA-binding of UPF2 (dashed red line). UPF3B Y160D mutation weakens complex formation of UPF3B with UPF2 (dashed red line). UPF3A isoform 2 does not bind UPF2 (solid red line). UPF3B and UPF3A compete (opposing solid red lines) for interaction with UPF2. UPF3B binds dsRNA via the RRM-L, NOPS-L, and CCL-1 domains (cyan, magenta and pink). RNA-induced oligomerization (bottom right) at high UPF3B concentrations is prevented by UPF2.

Our findings shed essential new light on three key roles of UPF3 in NMD:

Firstly, UPF3B binds mRNA and ribosomal subunits (19,20)*. In vitro*, UPF3B slows down translation termination and supports ribosome dissociation after peptide release (19). This activity requires the RRM-L and middle-domain of UPF3B, but not the EJC-binding motif (19). In agreement with its homology to paraspeckle proteins, we show that UPF3B undergoes RNA-induced oligomerization at high concentrations reliant on the RRM-L, NOPS-L and the coiled-coil domain (Figure 1C, Figure 6), congruent with coiled-coil regions being reported to be critical for paraspeckle formation (52). Consistent with ribosome-binding, UPF3B is a nucleo-cytoplasmic shuttling protein showing enrichment in nucleoli (25). In contrast, enrichment in nuclear or cytoplasmic granules is not reported so far, and it is unclear if cellular concentrations of UPF3B suffice to oligomerize on DNA or RNA. In support of mRNA-binding, UPF3B-RNA cross-linking studies showed that UPF3B interacts with mRNA 15-30 nucleotides upstream of exon-exon junctions (20) indicating that UPF3B’s mRNA binding may assist in positioning the NMD machinery. Interestingly, mRNA binding of UPF3A has recently been implicated in the genetic compensation response (61). While we characterize RNA-binding of UPF3B here, it is noteworthy that the sequence identity for the RRM-L-NOPS-L-CCL1 region is 67.3% between UPF3A and UPF3B, indicating that the molecular mechanisms of RNA binding and RNA-induced oligomerization are conserved for the two paralogs.

Secondly, the UPF2-UPF3B complex is essential for activation of UPF1 helicase (15,16) and of SMG1 kinase which phosphorylates UPF1 leading to recruitment of mRNA decay factors (17,18). Here, we show that the UPF2–UPF3B interface involves the NOPS-L region of the middle-domain composed of a linker and α-helix in addition to the interaction with the RRM-L domain of UPF3B described previously (21). The additional NOPS-L-mediated interactions lead to a 200-fold increase in affinity and thus are essential for high-affinity binding of UPF2.

Importantly, our UPF3B-UPF2 structure elucidates on the role of UPF3B’s neurodevelopmental disease-causing residue Y160 in stabilizing the UPF2-UPF3B complex (Figure 6). Y160 binds to a hydrophobic cleft of UPF2-MIF4GIII which previously was implicated in RNA-binding by UPF2 (21). Mutation to aspartate or phosphorylation of UPF3B’s Y160 residue displaces the residue from the hydrophobic cleft resulting in a ∼40-fold reduction in UPF2-binding affinity (Figure 4A), ultimately leading to reduced NMD efficiency and defects in neurite branching and plasticity (25,26).

In EMSAs, UPF2-UPF3B complex formation is favoured over UPF2–RNA and UPF3B–RNA binding/oligomerization (Figure 3F, Figure 6). We determine a considerably higher affinity between UPF2 and UPF3B (*K*_D_ = 0.5 nM) compared to each protein's affinity for RNA – we determined a *K*_D_ of 15 nM for UPF3B-dsRNA and a *K*_D_ of ∼700 nM for the UPF2-MIF4GIII-RNA complex (Figures 5A, 1B, Supplementary Figure S7F). Our data indicate a partial overlap in UPF3B binding sites for RNA and UPF2, both involving the RRM-L domain. This overlap likely is responsible for the switch we observe in EMSAs from RNA-induced oligomerization of UPF3B to a UPF2–UPF3B–RNA complex with defined stoichiometry (Figure 6). We therefore hypothesize that there are two different binding modes for RNA in UPF3B one in the absence and one in the presence of UPF2. Taken together, this highlights the importance of the middle-domain for UPF2-binding and suggests a switch from mRNA interaction (of both, the EJC-bound and the free UPF3B) to an NMD-activating UPF2–UPF3B complex.

In cells, weakening the UPF2-UPF3B interaction through UPF3B knockdown/knockout or UPF3B mutations in the RRM-L leads to upregulation of UPF3A levels (26,28,32). Consistently, the UPF3B Y160D mutation leads to an upregulation of UPF3A (25,26). This UPF3A upregulation appears to be achieved through stabilization of the inherently unstable free UPF3A protein via UPF2 binding (32). Unexpectedly, we found that UPF3A had a significantly higher affinity (∼10-fold) for UPF2 than UPF3B (Figure 5A), reliant on both small structural changes in a β-hairpin in the RRM-L, but mostly mediated by UPF3A’s C-terminal portion of the NOPS-L α-helical region which may support the formation of an additional polar interaction with UPF2 (Figure 5C–E).

Our findings raise the question as to how UPF3B can outcompete UPF3A for UPF2 binding in a direct competition in the cytosol. Cells could achieve this through differences in tissue-/developmental stage-dependent ratios of UPF3B and UPF3A, via phosphorylation of UPF3A and UPF3B at Y177 and Y160 respectively which impact the NOPS-L interaction with UPF2, and/or tissue-/developmental stage-dependent splicing of UPF3A (Figure 6) (24,32,34). Consistent with our finding that UPF3A isoform 2 does not interact with UPF2-MIF4GIII *in vitro*, loss of exon4 was shown to prevent UPF3A from rescuing NMD activity in UPF3B knockout cells (62). Isoform 2 was found to be transcribed in approximately a third of HEK293 cells in this study (62), while associated protein levels were undetectable. Splicing out the corresponding exon in UPF3B completely prevented UPF3B’s ability to rescue NMD in UPF3B and UPF3A double knockouts. Together, these results confirm that this region is essential for UPF3′s function in NMD and indicate splicing as a means of regulating UPF3A’s availability to bind UPF2.

Phosphorylation and splicing would enable cells to exploit the resulting changes in UPF2-binding affinity of the UPF3 paralogs to finetune NMD efficiency. Furthermore, it is conceivable that our analysis of pairwise interactions may not fully recapitulate what occurs in living cells. Interaction with further factors may additionally contribute to regulating access of UPF3s to UPF2, meriting future studies *in vivo* which explore contributions to protein stability and associated NMD activity of interaction partners such as nucleic acids or eRF3a; the latter was previously indicated as binding to the middle-domain (19). Finally, a ∼40-fold drop in *K*_D_ for UPF2 in the UPF3B Y160D mutant will lead to less complexes and an increased concentration of free UPF2 (and UPF3B Y160D) in the equilibrium. Free UPF2 could then bind and stabilize free UPF3A, resulting in the previously observed ∼3.5-fold upregulation of UPF3A levels in UPF3B knockout cells (26,32).

Thirdly, UPF3B’s role is believed to bridge UPF1-containing complexes to the EJC via the RRM-L and EJC-binding motif (EBM) respectively (15,17). In a UPF3B-deficient context, UPF3A can replace UPF3B as a weak NMD factor by binding to the EJC albeit with lower affinity (33). In mouse germ cells, UPF3A has been shown to even prevent NMD of an important set of mRNAs required for spermatogenesis (34). More recently, UPF3A was shown to act as an efficient NMD activator in human UPF3B knockout/knockdown cells (62,63). NMD was impaired only in double UPF3A and UPF3B knockouts (62,63). Interestingly in the UPF3B-deficient cells, NMD of a β-globin reporter mRNA with a PTC at codon 39 could not be rescued by UPF3A overexpression. The authors then exchanged UPF3A’s middle-domain with UPF3B’s middle-domain, which restored NMD of the reporter mRNA (63). Rescue experiments in UPF3-knockout/knockdown cells further support an important role of the middle-domain and a role of UPF3B in NMD which is independent from its bridging function (62): When UPF3B variants were expressed in a UPF3-depleted background, it was possible to delete individually the EBM, the middle-domain, or mutate the RRM-L virtually without reducing NMD. However, NMD was inhibited when any two of the three sites were altered (62), supporting our findings that both the middle-domain and RRM-L together are vital for high-affinity UPF2-binding, RNA-interaction and UPF3B’s role in translation termination (19).

Taken together, a revised view (Figure 6) of the importance and the role of UPF3′s domains is emerging, in which the middle-domain in particular plays a central role in NMD through its interactions with release factors, NMD substrate mRNAs, ribosomal subunits, and the MIF4GIII domain of UPF2.

## DATA AVAILABILITY

Coordinate and structure files have been deposited to the Protein Data Bank with accession code 7NWU for UPF3B and 7QG6 for UPF3A. All other data are available on request.

## Supplementary Material

gkac421_Supplemental_File
