# Supplementary material for: Structures of nonsense-mediated mRNA decay factors UPF3B and UPF3A in complex with UPF2 reveal molecular basis for competitive binding and for neurodevelopmental disorder-causing mutation
Source: Nucleic Acids Res. 2022 May 31;50(10):5934–47. doi: 10.1093/nar/gkac421 (PMC9177958; doi:10.1093/nar/gkac421)
Supplement: gkac421_Supplemental_File [file gkac421_supplemental_file.pdf]

Supplemental Material

Structures of nonsense-mediated mRNA decay factors UPF3B and UPF3A in complex with UPF2 reveal molecular basis for competitive binding and for neurodevelopmental disorder-causing mutation

Joshua C. Bufton, Kyle T. Powers, Jenn-Yeu A. Szeto, Christine Toelzer, Imre Berger, Christiane Schaffitzel

Correspondence to: christiane.berger-schaffitzel@bristol.ac.uk

This PDF file includes:

Supplemental Figures 1 to 13
Supplemental Tables 1 and 2

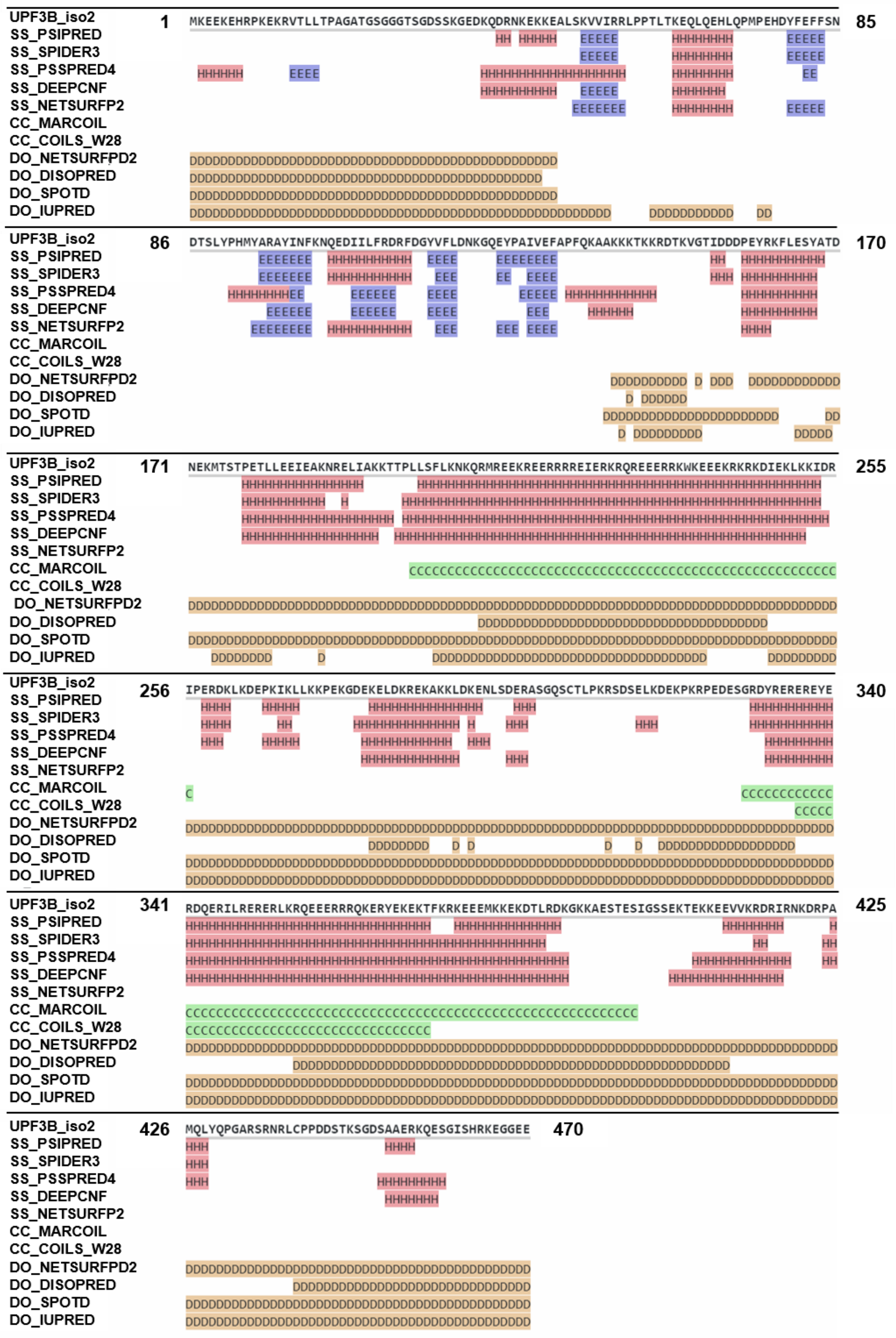
UPF3B_iso2
SS_PSIPRED
SS_SPIDER3
SS_PSSPRED4
SS_DEEPCNF
SS_NETSURFP2
CC_MARCOIL
CC_COILS_W28
DO_NETSURFPD2
DO_DISOPRED
DO_SPOTD
DO_IUPRED
1 MKEEKEHRPKEKRVTLLTPAGATGSGGGTSGDSSKGEDKQDRNKEKKEALSKVVIRRLPPTLTKEQLQEHLQPMPEHDYFEFFSN 85
86 DTSLYPHMYARAYINFKNQEDIILFRDRFDGYVFLDNKGQEYPAIVEFAPFQKAAKKKTKKRDTKVGTIDDDPEYRKFLESYATD 170
171 NEKMTSTPETLLEEIEAKNRELIAKKTTPLLSFLKNKQRMREEKREERRRREIERKRQREEERRKWKEEEKRKRKDIEKLKKIDR 255
256 IPERDKLKDEPKIKLLKKPEKGDEKELDKREKAKKLDKENLSDERASGQSCTLPKRSDSELKDEKPKRPEDESGRDYREREREYE 340
341 RDQERILRERERLKRQEEERRRQKERYEKEKTFKRKEEEMKKEKDTLRDKGKKAESTESIGSSEKTEKKEEVVKRDRIRNKDRPA 425
426 MQLYQPGARSRNRLCPPDDSTKSGDSAAERKQESGISHRKEGGEE 470

Supplemental Figure 1. UPF3B secondary structure prediction. Quick2D prediction (Max Planck Institute Bioinformatics Tool Kit) of full-length wildtype UPF3B (isoform2), covering several different secondary structure prediction servers (36). An overview of predicted secondary structure features including α-helices (red, H), β-sheets (blue E), coiled coils (green C) and disordered regions (brown D) is shown for each server underneath the primary protein sequence.

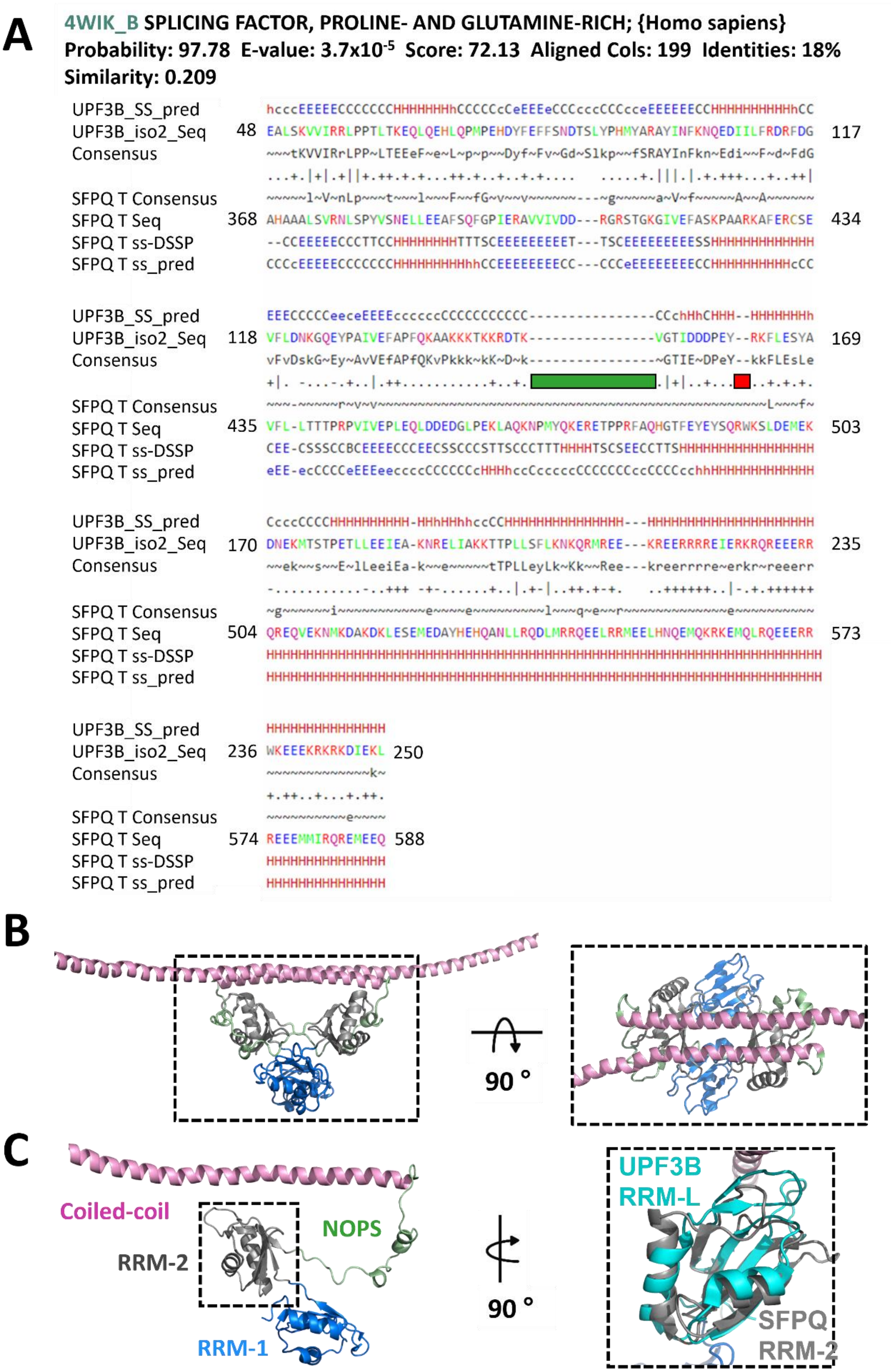
A
4WIK_B SPLICING FACTOR, PROLINE- AND GLUTAMINE-RICH; {Homo sapiens}
Probability: 97.78 E-value: 3.7x10-5 Score: 72.13 Aligned Cols: 199 Identities: 18%
Similarity: 0.209
UPF3B_SS_pred
UPF3B_iso2_Seq
Consensus
SFPQ T Consensus
SFPQ T Seq
SFPQ T ss-DSSP
SFPQ T ss_pred
B
90 °
C
Coiled-coil
NOPS
RRM-2
RRM-1
90 °
UPF3B
RRM-L
SFPQ
RRM-2

Supplemental Figure 2. SFPQ HHpred homology detection hit for UPF3B. (**A**) UPF3B Isoform2 HHpred homology detection hit alignment (36) for PDB ID 4WIK (SFPQ-369-598 homodimer) (48). The probability value describes the likelihood of the hit having homology to the query. The E-value describes how many hits with a better probability score would be expected if the database contained only unrelated hits (36). Homology between the sequences is indicated by the matching of the consensus sequences and by the presence of conserved motifs marked by '+' and '|' signs between alignments. Green and red bars denote the locations of the UPF3B "deletions" in proximity to SFPQ residues Y490 and W494, which are implicated in dimerisation of DBHS family members (48). (**B**) Side (left) and top (right) views of the crystal structure of homodimeric SFPQ-276–598 (PDB ID: 4WIJ) (48). (**C**) Left: Monomer of SFPQ-276–598 highlighting DBHS family core domains. The N-terminal tandem RRM1-RRM2 folds are depicted in blue and grey, respectively. The NOPS domain is coloured in light green and the antiparallel coiled-coil domain in pink. Right: Superimposition of UPF3B's RRM-L domain (1UW4, cyan) (21) onto the RRM-2 domain of SFPQ indicating a high degree of homology.

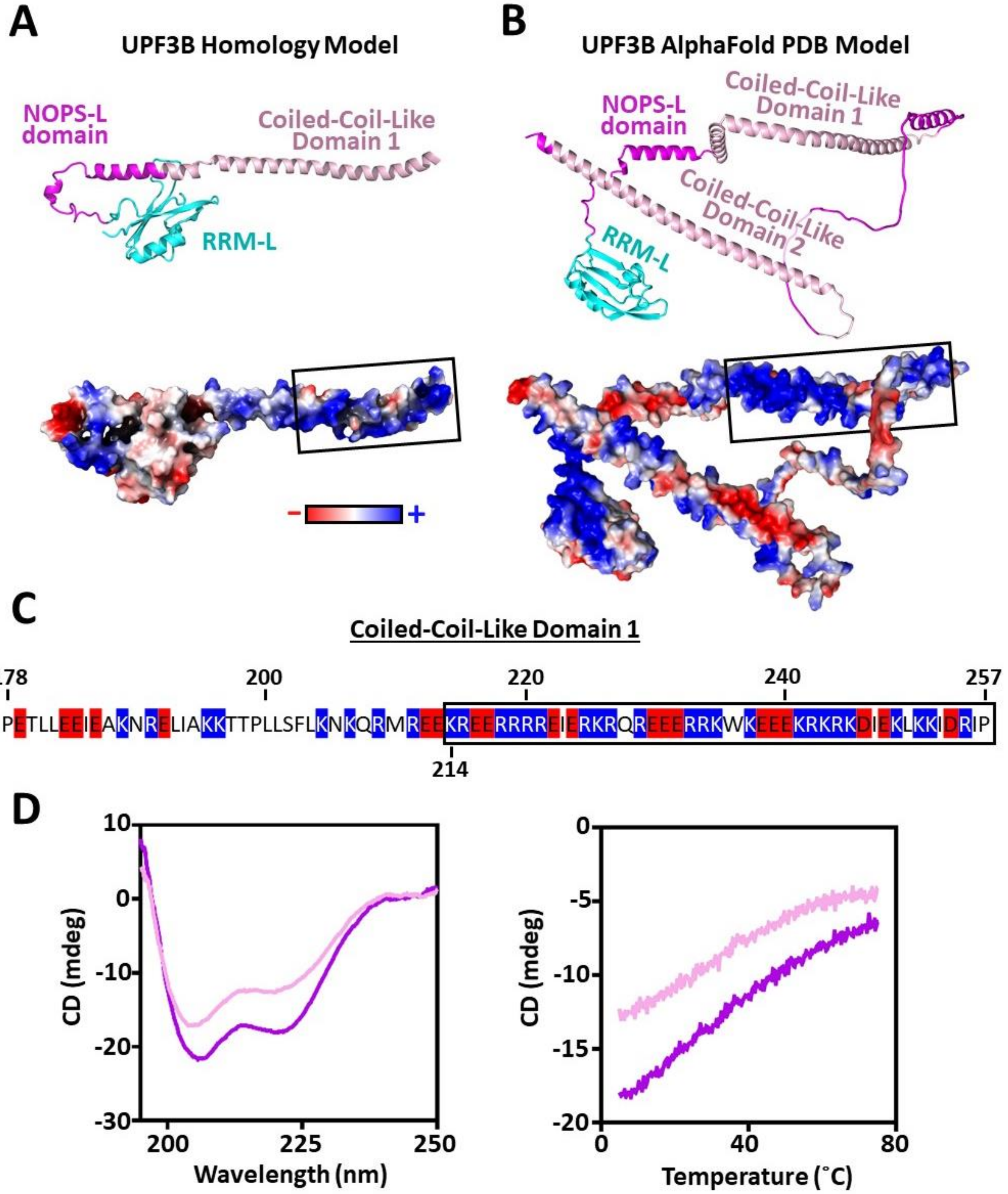

Supplemental Figure 3. HHpred homology model, AlphaFold model, high-affinity RNA binding sequence and CD Spectra of UPF3B. (**A**) Above: UPF3B homology model created with MODELLER using the structure of DBHS protein SFPQ (PDBID:4WIK) as a template (48). UPF3B's RRM-L is coloured in cyan, predicted NOPS-like domain (NOPS-L) in magenta and coiled-coil-like domain in light pink. Below: Surface

representation of the UPF3B homology model indicating electrostatic potential; positive (blue), neutral (white) and negative (red). The map was produced using the Adaptive Poisson-Boltzmann Solver within PyMol. (**B**) Above: Full length UPF3B model generated by AlphaFold predictive software. UPF3B's RRM-L is coloured in cyan (model confidence >90%), predicted NOPS-like domain (NOPS-L) in magenta and coiled-coil-like domains in light pink (model confidence: 70-90%). Below: Surface representation of the UPF3B AlphaFold model indicating electrostatic potential; positive (blue), neutral (white) and negative (red). The map was produced using the Adaptive Poisson-Boltzmann Solver within PyMol. (**C**) The sequence of the coiled-coil-like domain 1 is shown. Positively or negatively charged residues are highlighted in blue or red, respectively. The black boxes in panels A, B and C indicate the part of the CCL-1 domain required for high-affinity RNA binding by UPF3B. (**D**) Left: CD spectroscopy trace of a 195-250 nm wavelength scan for UPF3B-146-256 (light pink line) and UPF3B-146-417 (magenta line) middle-domain fragments revealing a double dip trace indicative of α-helical structure. Right: temperature melt of the same constructs from 5-75°C taking CD measurements at 222 nm indicating a non-sigmoidal curve.

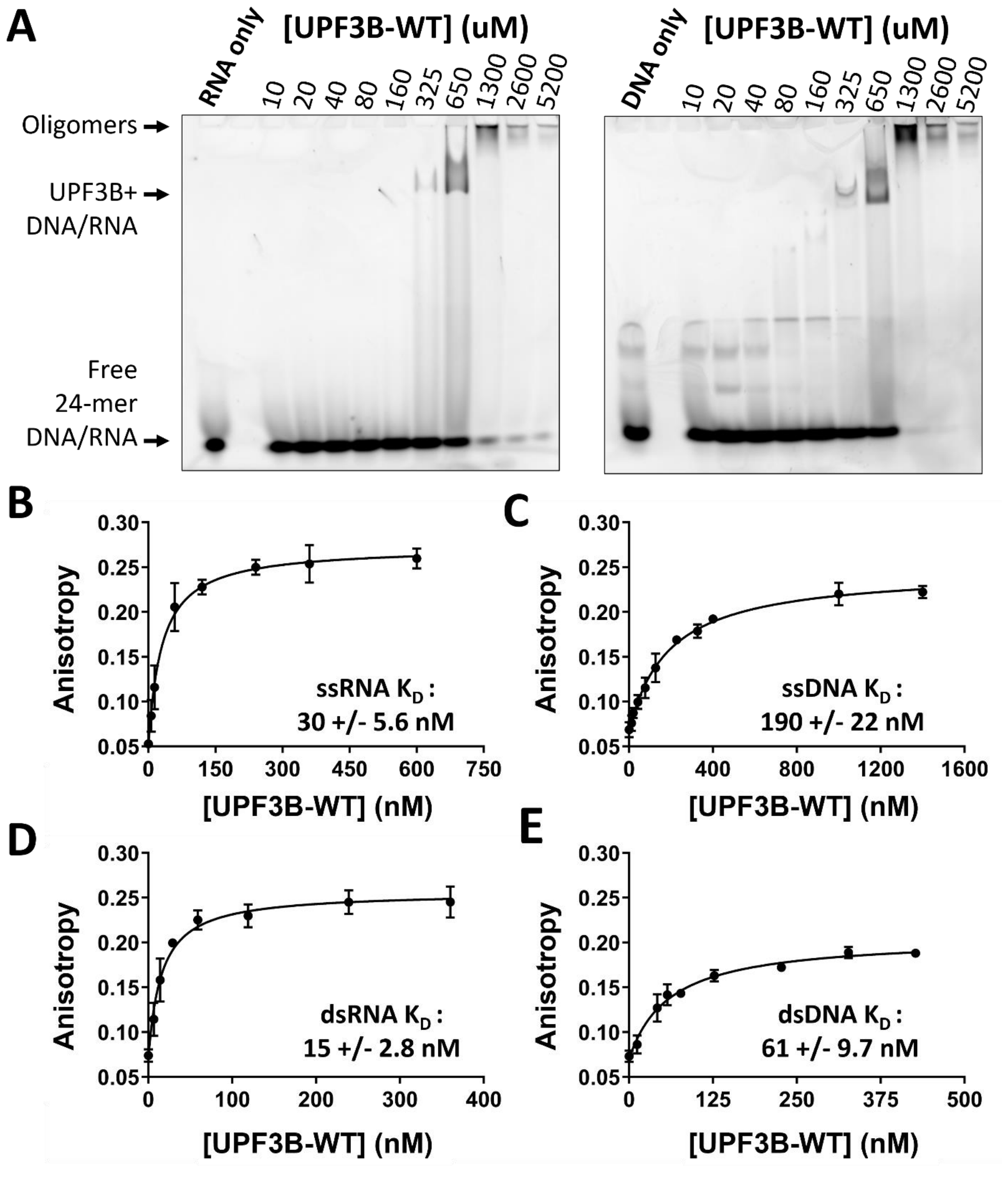
A
RNA only
[UPF3B-WT] (uM)
10 20 40 80 160 325 650 1300 2600 5200
DNA only
[UPF3B-WT] (uM)
10 20 40 80 160 325 650 1300 2600 5200
Oligomers
UPF3B+ DNA/RNA
Free 24-mer DNA/RNA
B
Anisotropy
ssRNA K_D : 30 +/- 5.6 nM
[UPF3B-WT] (nM)
C
Anisotropy
ssDNA K_D : 190 +/- 22 nM
[UPF3B-WT] (nM)
D
Anisotropy
dsRNA K_D : 15 +/- 2.8 nM
[UPF3B-WT] (nM)
E
Anisotropy
dsDNA K_D : 61 +/- 9.7 nM
[UPF3B-WT] (nM)

Supplemental Figure 4. UPF3B nucleic acid-induced oligomerisation and substrate preference. (**A**) Electro-mobility-shift-assays (EMSAs) of UPF3B-WT with single-stranded 24-mer RNA (left) and single-stranded 24-mer DNA (right) indicating oligomerisation behaviour of UPF3B in the presence of both ssDNA and ssRNA. Representative gel image shown of independent triplicate EMSAs conducted. The concentration of RNA/DNA probe required for band shift detection was 250 nM due to limitations in detecting multiple shifted products at lower nucleic acid concentrations. (**B-E**) Fluorescence anisotropy binding curves for UPF3B-WT with 24-mer ssRNA (**B**), ssDNA (**C**), dsRNA (**D**) and dsDNA (**E**) indicating a preference for RNA over DNA, as well as double-stranded oligonucleotides over single-stranded oligonucleotides. Protein titrations and measurements were carried out in triplicate to produce error bars by standard deviation before fitting a single component binding equation (see methods) in GraphPad Prism to calculate the reported dissociation constants highlighted in each graph. Sequences of the oligomers are listed in Supplemental Table 1.

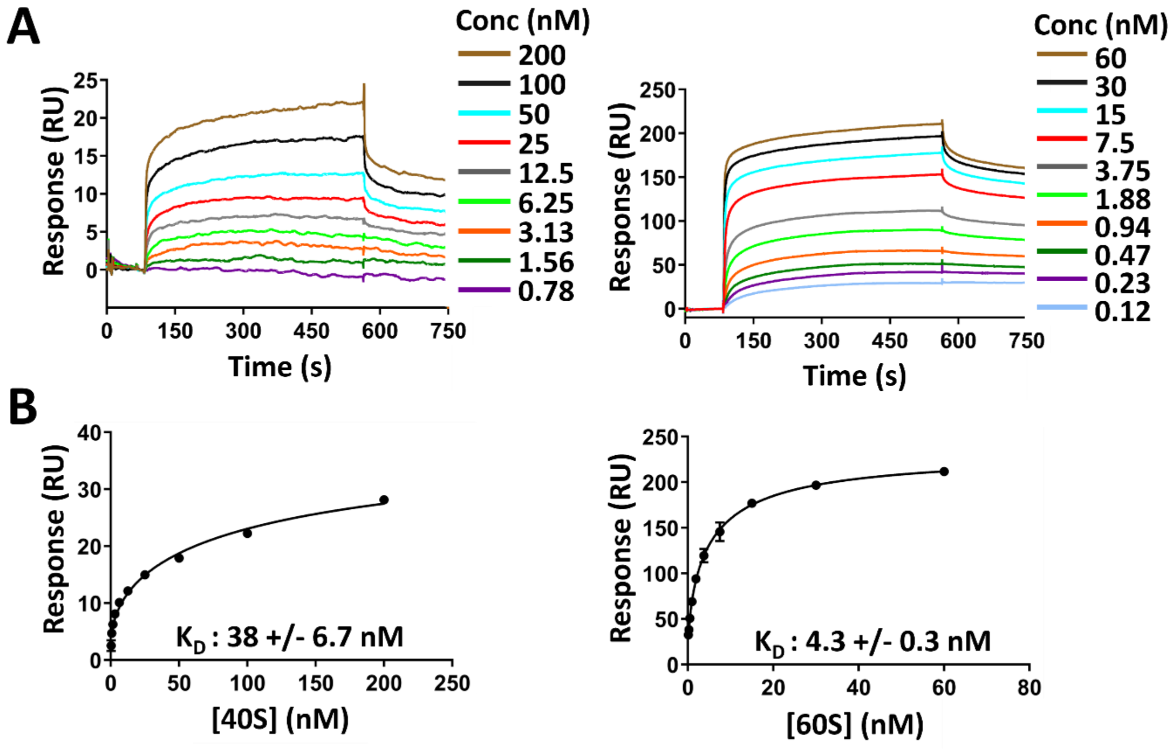

Supplemental Figure 5. Human 40S and 60S ribosomal subunit binding of UPF3B. (**A**) Representative sensorgrams for each analyte concentration of 40S (left) and 60S (right) subunits injected over immobilised biotinylated Avi-UPF3B-WT. (**B**) Steady-state fit plots generated by plotting responses produced by the small ribosomal subunit 40S (left) and the large ribosomal subunit 60S (right) at 450 seconds post injection vs concentration of analyte. Data points were fitted with the single component binding equation in GraphPad Prism to calculate the reported K_D values.

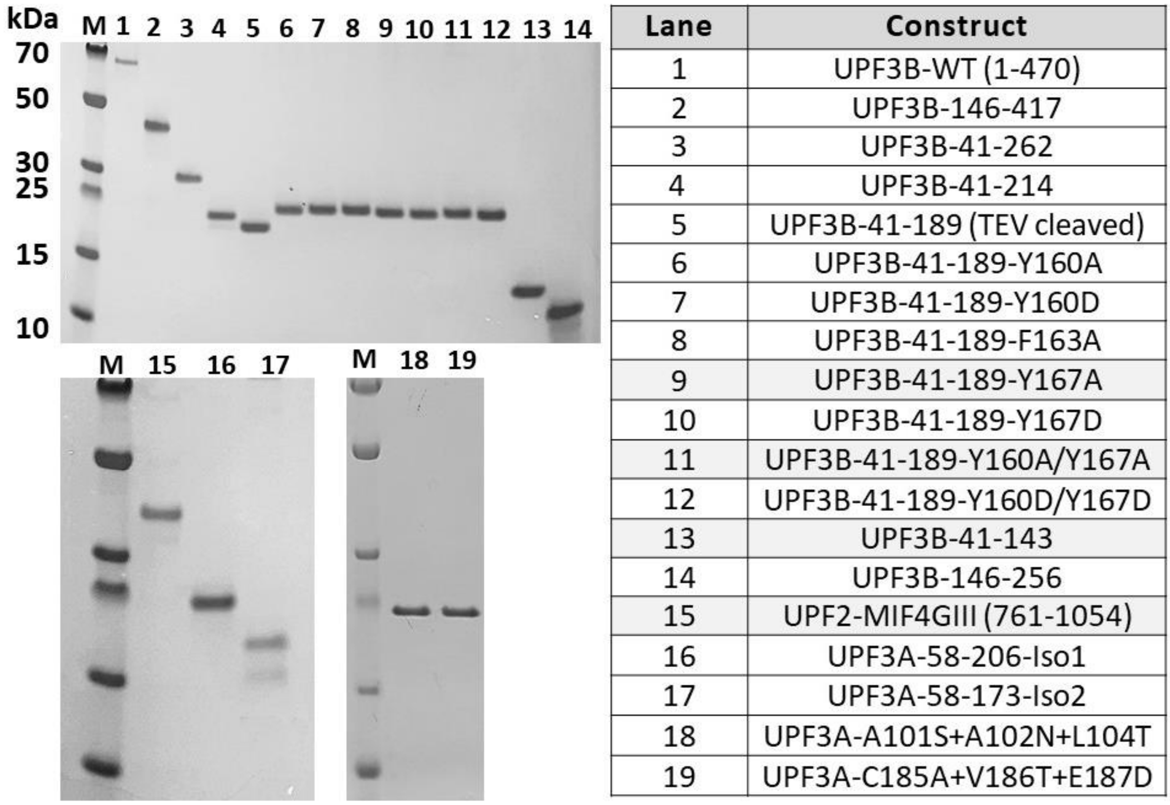

Lane	Construct
1	UPF3B-WT (1-470)
2	UPF3B-146-417
3	UPF3B-41-262
4	UPF3B-41-214
5	UPF3B-41-189 (TEV cleaved)
6	UPF3B-41-189-Y160A
7	UPF3B-41-189-Y160D
8	UPF3B-41-189-F163A
9	UPF3B-41-189-Y167A
10	UPF3B-41-189-Y167D
11	UPF3B-41-189-Y160A/Y167A
12	UPF3B-41-189-Y160D/Y167D
13	UPF3B-41-143
14	UPF3B-146-256
15	UPF2-MIF4GIII (761-1054)
16	UPF3A-58-206-Iso1
17	UPF3A-58-173-Iso2
18	UPF3A-A101S+A102N+L104T
19	UPF3A-C185A+V186T+E187D

Supplemental Figure 6. SDS-PAGE of purified protein samples used in this study. Coomassie-stained 4-12% NuPAGE Bis-Tris SDS-PAGE gels of the protein samples produced in this study loaded at ~0.5 µg. M stands for molecular weight marker.

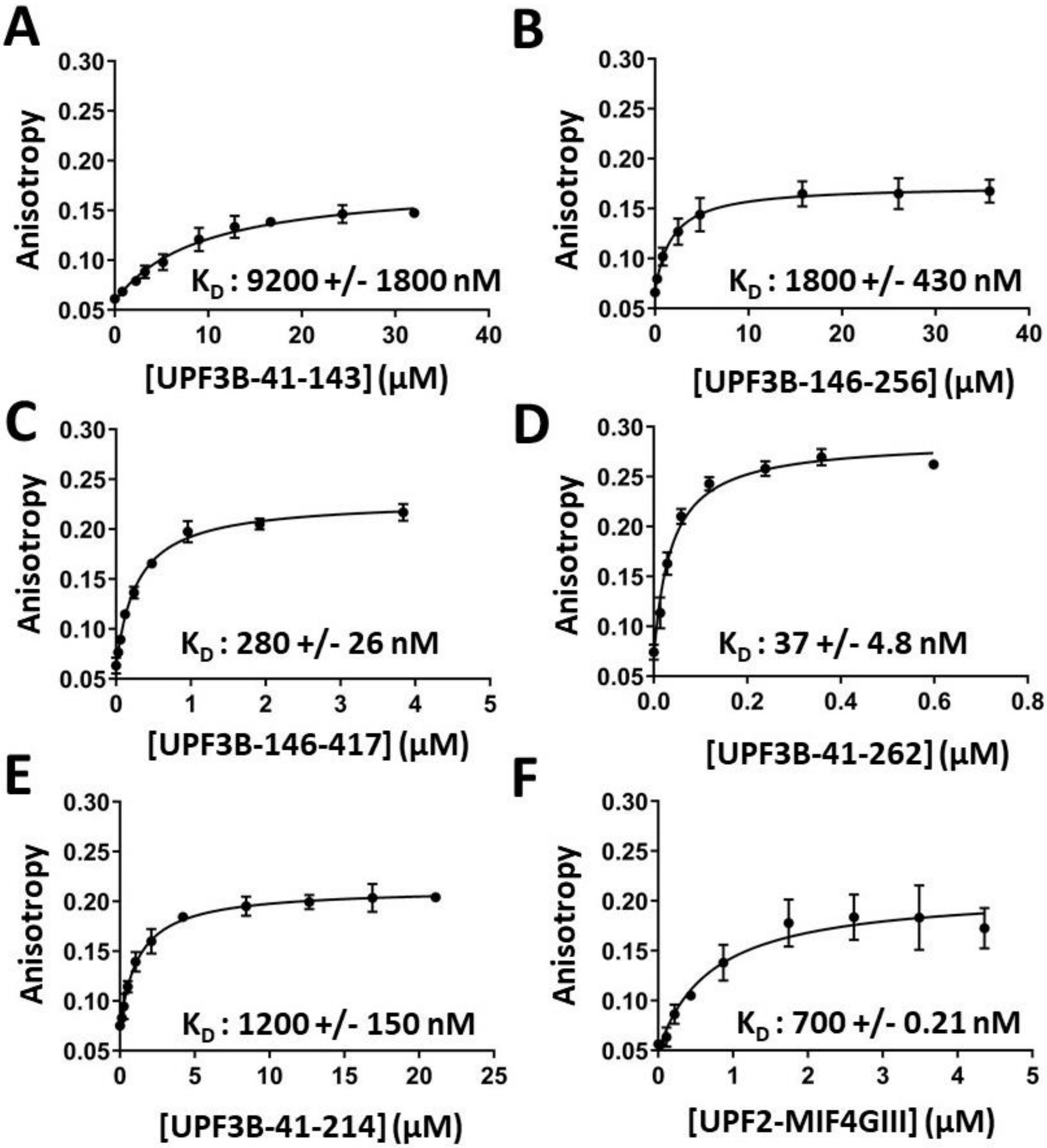

Supplemental Figure 7. RNA binding of UPF3B variants and of UPF2-MIF4GIII. Fluorescence anisotropy binding curves for UPF3B-41-143 (**A**), UPF3B-146-256 (**B**), UPF3B-146-417 (**C**), UPF3B-41-262 (**D**), and UPF3B-41-214 (**E**) titrated against 24-mer HEX-labelled dsRNA. Binding curve for UPF2-MIF4GIII (**F**) vs 24-mer HEX-labelled ssRNA. Protein titrations were carried out in triplicate and error bars plotted via standard deviation before fitting using the single component binding equation in GraphPad Prism to calculate the reported dissociation constants.

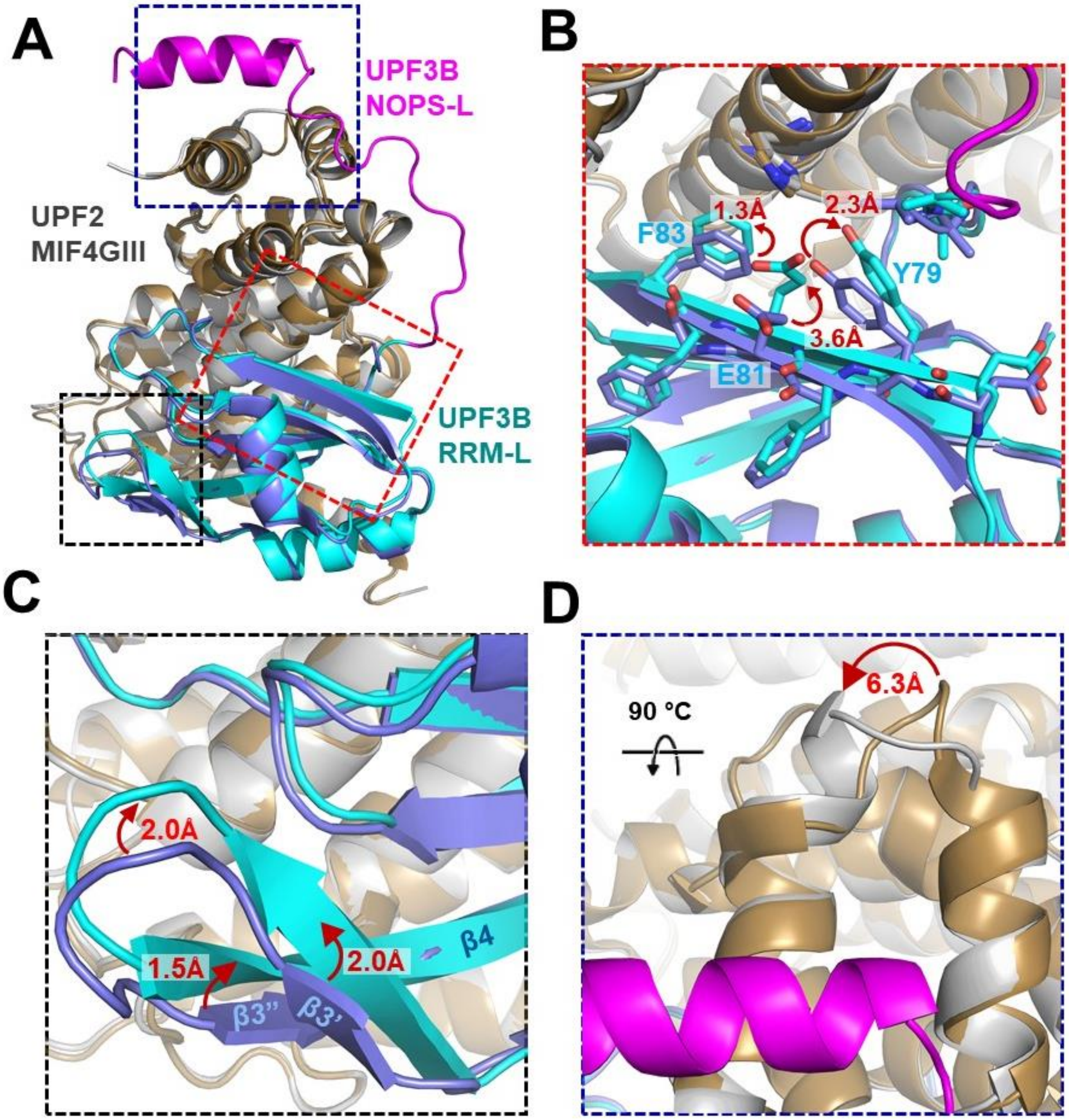

Supplemental Figure 8. Comparison of the UPF3B-41-189+UPF2-MIF4GIII co-crystal structure from this study with the crystal structure of UPF3B RRM-L+UPF2-MIF4GIII (PDB ID 1UW4) (21)**.** UPF3B is coloured cyan (RRM-L) and magenta (NOPS-L) (our structure), and light blue (RRM-L, PDB ID 1UW4); UPF2-MIF4GIII is grey (our structure) and gold (PDB ID 1UW4). (**A**) Alignment of the two crystal structures. (**B**) Zoom in on the interface region comprising the β2-strand of UPF3B (highlighted with a red box in panel A). (**C**) Zoom in on β3', β3", and β4 (1UW4) and continuous sheets β3'/3" and β-4 (our structure) of UPF3B (highlighted with a black box in panel A). (**D**) Zoom in on helices α-1 and α-2 of UPF2 (highlighted with a blue box in panel A).

```
Homo-sapiens-UPF3B                   KTKKRDTKVGTIDDDPEYRKFLESYATD
Pan-troglodytes-UPF3B                KTKKRDTKVGTIDDDPEYRKFLESYATD
Macaca-mulatta-UPF3B                 KTKKRDTKVGTIDDDPEYRKFLESYATD
Bos-taurus-UPF3B                     KTKKRDTKVGTIDDDPEYRKFLESYAAD
Canis-lupus-familiaris-UPF3B         KTKKRDTKVGTIDDDPEYRKFLESYAAD
Rattus-norvegicus-UPF3B              KIKKRDTKVGTIEDDPEYRKFLESYATD
Mus-musculus-UPF3B                   KIKKRDTKVGTIEDDPEYRKFLESYATD
Tetraodon-nigroviridis-UPF3B         RSKKRDAKCGTINEDPEYKKFLEFYNGD
Danio-rerio-UPF3                     RSKKKDAKSGTIDDDADYKKFLEFYNGD
Taeniopygia-guttata-UPF3             KSKKKDAKTGTIEDDPEYKKFLESYSAD
Gallus-gallus-UPF3                   KSKKKDAKTGTIEDDPEYKKFLESYSAD
Xenopus-laevis-UPF3                  KSKKQDSKIGTIDEDPEYRKFLDSYTMD
Homo-sapiens-UPF3A                   KLRKKDAKTGSIEDDPEYKKFLETYCVE
Pan-troglodytes-UPF3A                KLRKKDAKTGSIEDDPEYKKFLETYWVE
Macaca-mulatta-UPF3A                 KLKKKDAKTGSIEDDPEYKKFLETYCVE
Bos-taurus-UPF3A                     KLKKKDAKTGSIEDDPEYKKFLETYCVE
Canis-lupus-familiaris-UPF3A         KLKKKDAKTGSIEDDPEYKKFLETYCVE
Rattus-norvegicus-UPF3A              KVKKKDAKTGSIEDDPEYKQFLESYSLE
Mus-musculus-UPF3A                   KLKKKDAKTGSIEDDPEYKQFLESYSLE
Tetraodon-nigroviridis-UPF3A         KLKKKDAKAGSIEEDPEYKRFLENISCD
Strongylocentrotus-purpuratus-UPF3B  IGKKVDARTATIEEDSDYKKFVETLNAE
Drosophila-melanogaster-UPF3         KARNDDSKVNTIESEPHYQEFIKRLAQE
Caenorhabditis-elegans               NRMKEDTRVGAILTDKYYLDFCKKLEEE
Oryza-sativa-UPF3                    -NTKKDARQGTIMKDPEYLEFLESISKP
Arabidopsis-thaliana-UPF3            -SDKKDPREGSISKDPDYLEFLKVIAQP
Schistosoma-japonicum-UPF3A          KRDKVDKKQGSLLGDSEYIEFVKSMESA
                                        : * :  ::  :  *  * .
```

Supplemental Figure 9. UPF3 alignments of NOPS-L region. Homologous sequences obtained through submission of full-length human UPF3B isoform2 to NCBI BLAST (56) before aligning using EBI Clustal Omega multiple sequence alignment tool (57). The alignment is shown for human UPF3B residues 143-170, corresponding to the NOPS-L domain. An asterisk (*) indicates conserved residues in the alignment, a colon (:) indicates residues sharing conserved properties, while a period (.) indicates residues with weakly similar properties.

160 167

146 KRDTKVGTIDDDPEYRKFLESYATDNEKMTSTPETLLEEIEAKNRELIAKKTTPLLSFLK 205

206 NKQRMREEKREERRRREIERKRQREEERRKWKEEEKRKRKDIEKLKKIDRIPERDKLKDE 265

310

266 PKIKLLKKPEKGDEKELDKREKAKKLDKENLSDERASGQSCTLPKRSDSELKDEKPKRPE 325

326 DESGRDYREREREYERDQERILRERERLKRQEEERRRQKERYEKEKTFKRKEEEMKKEKD 385

396 399 403

386 TLRDKGKKAESTESIGSSEKTEKKEEVVKRDR 417

Peptide	Identified PTM sites	Probability of PTM at each site
VGTIDDDPEYRK	Y10(Phospho)	T(3): 0.0; Y(10): 100.0
FLESYATDNEK	Y5(Phospho)	S(4): 0.5; Y(5): 99.5; T(7): 0.0
FLESYATDNEK	Y5(Phospho)	S(4): 6.3; Y(5): 93.2; T(7): 0.5
LDKENLSDER	S7(Phospho)	S(7): 100.0
LDKENLSDER	S7(Phospho)	S(7): 100.0
LDKENLSDER	S7(Phospho)	S(7): 100.0
LDKENLSDER	S7(Phospho)	S(7): 100.0
LDKENLSDER	S7(Phospho)	S(7): 100.0
ENLSDERASGQSCTLPK	S4(Phospho);	S(4): 100.0; S(9): 0.0; S(12): 0.0; T(14): 0.0
ENLSDERASGQSCTLPK	S4(Phospho);	S(4): 100.0; S(9): 0.0; S(12): 0.0; T(14): 0.0
ENLSDERASGQSCTLPK	S4(Phospho);	S(4): 100.0; S(9): 0.0; S(12): 0.0; T(14): 0.0
KAESTESIGSSEK	S4(Phospho); S7(Phospho)	S(4): 89.3; T(5): 12.1; S(7): 98.6; S(10): 0.0; S(11): 0.0
KAESTESIGSSEK	S4(Phospho); S7(Phospho)	S(4): 90.3; T(5): 9.8; S(7): 99.8; S(10): 0.0; S(11): 0.0
KAESTESIGSSEK	S4(Phospho); S7(Phospho)	S(4): 91.1; T(5): 9.0; S(7): 99.9; S(10): 0.0; S(11): 0.0
KAESTESIGSSEK	S4(Phospho); S7(Phospho)	S(4): 99.1; T(5): 1.0; S(7): 99.9; S(10): 0.0; S(11): 0.0
KAESTESIGSSEK	S4(Phospho); S7(Phospho)	S(4): 89.3; T(5): 12.1; S(7): 98.6; S(10): 0.0; S(11): 0.0
KAESTESIGSSEK	S4(Phospho); S7(Phospho)	S(4): 90.4; T(5): 19.1; S(7): 90.4; S(10): 0.0; S(11): 0.0
KAESTESIGSSEK	S4(Phospho); S7(Phospho)	S(4): 98.9; T(5): 10.8; S(7): 90.4; S(10): 0.0; S(11): 0.0
KAESTESIGSSEK	S4(Phospho); S7(Phospho)	S(4): 54.5; T(5): 54.5; S(7): 90.3; S(10): 0.5; S(11): 0.3
KAESTESIGSSEK	S4(Phospho); S7(Phospho)	S(4): 50.4; T(5): 50.4; S(7): 99.1; S(10): 0.0; S(11): 0.0
KAESTESIGSSEK	S4(Phospho); S7(Phospho)	S(4): 98.6; T(5): 12.1; S(7): 89.3; S(10): 0.0; S(11): 0.0
KAESTESIGSSEK	S4(Phospho); S7(Phospho)	S(4): 98.6; T(5): 2.9; S(7): 98.6; S(10): 0.0; S(11): 0.0
KAESTESIGSSEK	S4(Phospho); S7(Phospho)	S(4): 87.2; T(5): 25.5; S(7): 87.2; S(10): 0.0; S(11): 0.0
KAESTESIGSSEK	S4(Phospho)	S(4): 90.3; T(5): 9.7; S(7): 0.0; S(10): 0.0; S(11): 0.0
KAESTESIGSSEK	T5(Phospho); S7(Phospho)	S(4): 87.2; T(5): 25.5; S(7): 87.2; S(10): 0.0; S(11): 0.0
AESTESIGSSEK	S3(Phospho); S6(Phospho)	S(3): 99.9; T(4): 97.8; S(6): 2.3; S(9): 0.0; S(10): 0.0
AESTESIGSSEK	S3(Phospho); S6(Phospho)	S(3): 89.5; T(4): 20.9; S(6): 89.5; S(9): 0.0; S(10): 0.0
AESTESIGSSEK	S3(Phospho)	S(3): 98.9; T(4): 1.1; S(6): 0.0; S(9): 0.0; S(10): 0.0
AESTESIGSSEK	S3(Phospho)	S(3): 98.5; T(4): 1.5; S(6): 0.0; S(9): 0.0; S(10): 0.0
AESTESIGSSEK	S9(Phospho)	S(3): 0.0; T(4): 0.0; S(6): 0.0; S(9): 9.8; S(10): 90.2
AESTESIGSSEK	S3(Phospho); S6(Phospho)	S(3): 97.3; T(4): 5.3; S(6): 96.3; S(9): 0.5; S(10): 0.5; T(13): 0.0
AESTESIGSSEK	S3(Phospho)	S(3): 98.0; T(4): 2.0; S(6): 0.0; S(9): 0.0; S(10): 0.0; T(13): 0.0

Supplemental Figure 10. Phosphorylation mapping mass spectrometry of human UPF3B. Data collected using purified full-size UPF3B expressed using the MultiBac insect cell expression system. The identified peptide fragments are highlighted in light blue, purple, orange, and red for peptides VGTIDDDPEYRK, FLESYATDNEK, ENLSDERASGQSCTLPK and AESTESIGSSEK, respectively. These peptides were identified as having multiples of +80 mass indicative of phosphorylation sites and a second fragmentation step identified residues with a more than 90% probability of harbouring the phosphorylation.

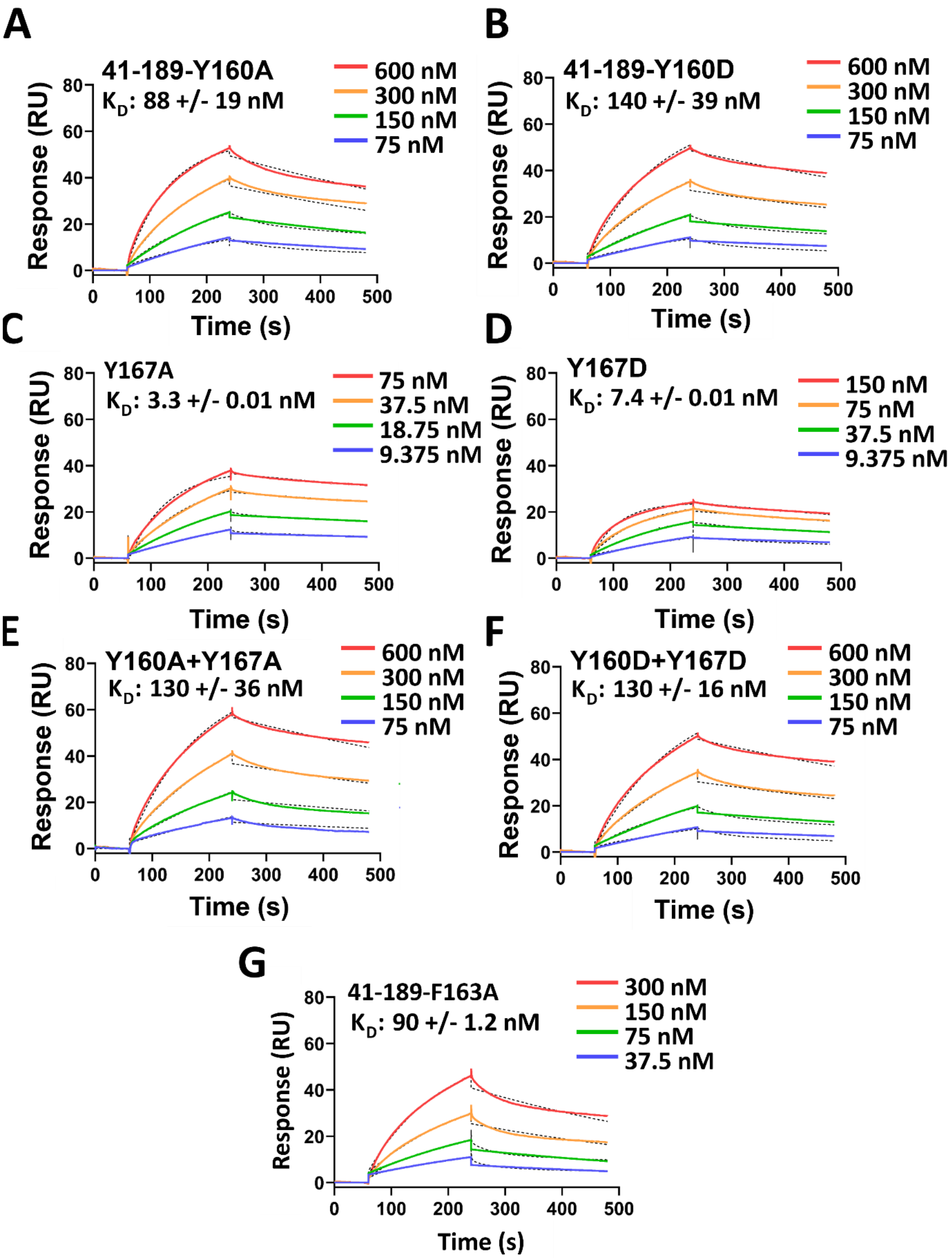
A
41-189-Y160A
K_D: 88 +/- 19 nM
600 nM
300 nM
150 nM
75 nM
Response (RU)
Time (s)
B
41-189-Y160D
K_D: 140 +/- 39 nM
600 nM
300 nM
150 nM
75 nM
Response (RU)
Time (s)
C
Y167A
K_D: 3.3 +/- 0.01 nM
75 nM
37.5 nM
18.75 nM
9.375 nM
Response (RU)
Time (s)
D
Y167D
K_D: 7.4 +/- 0.01 nM
150 nM
75 nM
37.5 nM
9.375 nM
Response (RU)
Time (s)
E
Y160A+Y167A
K_D: 130 +/- 36 nM
600 nM
300 nM
150 nM
75 nM
Response (RU)
Time (s)
F
Y160D+Y167D
K_D: 130 +/- 16 nM
600 nM
300 nM
150 nM
75 nM
Response (RU)
Time (s)
G
41-189-F163A
K_D: 90 +/- 1.2 nM
300 nM
150 nM
75 nM
37.5 nM
Response (RU)
Time (s)

Supplemental Figure 11. SPR sensorgrams of UPF3B-41-189 variants binding to immobilised UPF2-MIF4GIII. Representative sensorgrams for 4 analyte concentrations per UPF3B mutant are shown and their corresponding fits (black dotted lines). Measurements were performed in triplicate. Fits were globally fitted with the 1:1 binding model within the T200 Biacore Evaluation Software to calculate indicated K_D values. Sensorgrams are shown for UPF3B-41-189-Y160A (**A**), UPF3B-41-189-Y160D (**B**), UPF3B-41-189-Y167A (**C**), UPF3B-41-189-Y167D (**D**), UPF3B-41-189-Y160A+Y167A (**E**), UPF3B-41-189-Y160D+Y167D (**F**), and UPF3B-41-189-F163A (**G**).

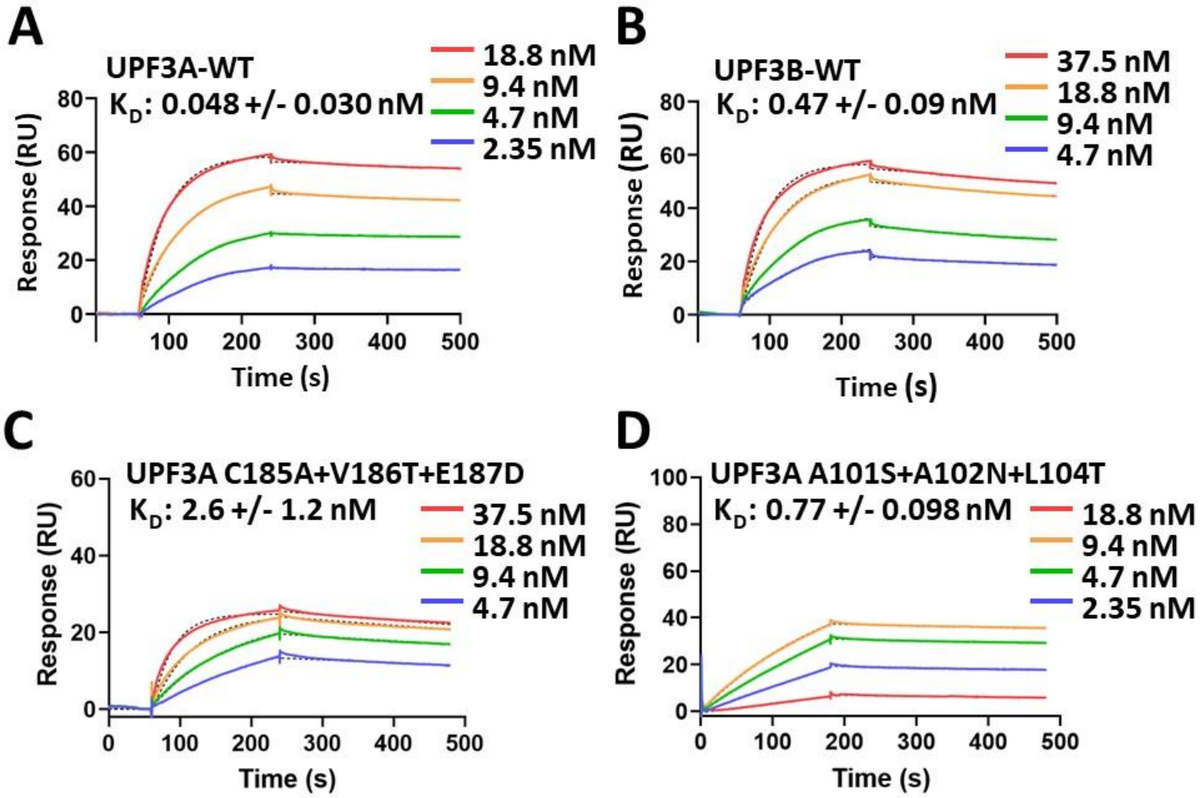

Supplemental Figure 12. SPR sensorgrams of wildtype UPF3A and UPF3B and UPF3A-to-UPF3B mutation variants binding to immobilised UPF2-MIF4GIII. Representative sensorgrams for four analyte concentrations of (**A**) UPF3A-WT**,** (**B**) UPF3B-WT**,** (**C**) UPF3A-58-206-NOPS* (C185A-V186T-E187D)**,** and (**D**) UPF3A-58-206-RRM* (A101S-A102N-L104T) binding to immobilised UPF2-MIF4GIII. Measurements were performed in triplicate. Each concentration was individually fitted with the 1:1 binding model within the T200 Biacore Evaluation Software to calculate indicated K_D values.

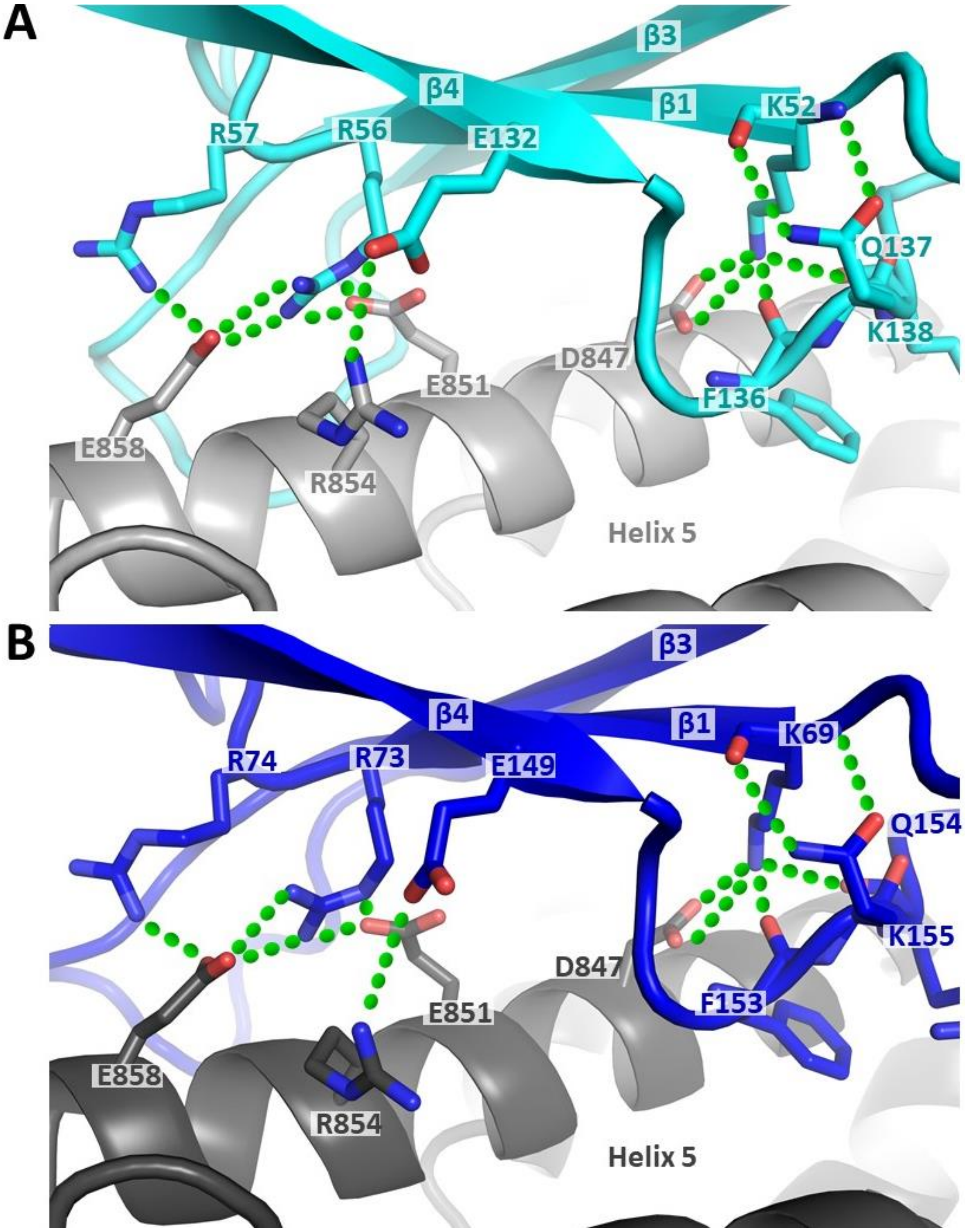

Supplemental Figure 13. Comparison of the UPF3B-41-189+UPF2-MIF4GIII crystal structure with the crystal structure of UPF3A-58-206+UPF2-MIF4GIII. UPF3B is coloured cyan (RRM-L) and UPF3A's corresponding region is coloured dark blue; UPF2-MIF4GIII is grey (UPF3B-UPF2 structure) and dark grey (UPF3A-UPF2 structure). (**A**) UPF3B and UPF2 residues involved in protein-protein interaction (green dots) are indicated. (**B**) UPF3A and UPF2 residues involved in protein-protein interaction (green dots) are indicated.

Hex-Oligos	Sequence
HEX-24mer-ssDNA	5’ HEX-CCC TGA GCT GAC GCA GCA CCT GGG 3’
HEX-24mer-ssRNA	5’ HEX-CAC UGA UCU GAC GCU GCA CCU GGG 3’
HEX-24mer-dsDNA:	5’ HEX-CCC TGA GCT GAC GCA GCA CCT GGG<br>GGG ACT CGA CTG CGT CGT GGA CCC
HEX-24mer-dsRNA	5’ HEX- CCC UGA GCU GAC GCA GCA CCU GGG<br>GGG ACU CGA CUG CGU CGU GGA CCC

Supplemental Table 1. HEX-oligo sequences used for fluorescence anisotropy and EMSA experiments.

Cloning Primers	Sequence
UPF3B.41.Forward	5’ ACT CGC CAT GGA TCG CAA CAA GGA GAA GAA 3’
UPF3B.143.Reverse	5’ GGA CGT CGA CTT ACT TCT TTT TTG CAG CTT TTT G 3’
UPF3B.262.Reverse	5’ GGA CGT CGA CTT ATA ATT TGT CCC TTT CTG GA 3’
UPF3B.189.Reverse	5’ GGA CGT CGA CTT AAT TTT TTG CTT CTA TTT CCT CTA GC 3’
UPF3B.214.Reverse	5’ GGACGTCGACTTACTTTTCTTCTCTCATTCTCTGC 3’
**Q5 Site Directed Mutagenesis Primers**	**Sequence**
UPF2-MIF4GIII-Avi-insert.Forward	5' GCA GAA AAT TGA ATG GCA TGA AGA TTA CGA TAT CCC AAC G 3'
UPF2-MIF4GIII-Avi-insert.Reverse	5' GCT TCA AAA ATA TCG TTC AGG CCG TGA TGG TGA TGG TGA TG 3'
UPF3B-Y160D.Forward	5’ TGA TCC AGA AGA TAG AAA GTT TTT GG 3’
UPF3B-Y160D.Reverse	5’ TCA TCG ATA GTC CCG ACT TTG 3’
UPF3B-Y160A.Forward	5’ TGA TCC AGA AGC TAG AAA GTT TTT GGA AAG TTA TGC 3’
UPF3B-Y160A.Reverse	5’ TCA TCG ATA GTC CCG ACT TTG 3’
UPF3B-F163A.Forward	5’ ATA TAG AAA GGC ATT GGA AAG TTA TGC CAC 3’
UPF3B-F163A.Reverse	5’ TCT GGA TCA TCA TCG ATA G 3’
UPF3B-Y167D.Forward	5’ TTT GGA AAG TGA TGC CAC AGA CA 3’
UPF3B-Y167D.Reverse	5’ AAC TTT CTA TAT TCT GGA TCA TCA TC 3’
UPF3B-Y167A.Forward	5’ TTT GGA AAG TGC TGC CAC AGA CAA TG 3’
UPF3B-Y167A.Reverse	5’ AAC TTT CTA TAT TCT GGA TCA TC 3’
UPF3B-Y160D-167D.Forward	5’ TTG GAA AGT GAT GCC ACA GAC AAT GAG AAA ATG 3’
UPF3B-Y160D-167D.Reverse	5’ AAA CTT TCT ATC TTC TGG ATC ATC ATC GAT AG 3’
UPF3B-Y160A-167A.Forward	5’ TTG GAA AGT GCT GCC ACA GAC AAT GAG AAA ATG 3’
UPF3B-Y160A-167A.Reverse	5’ AAA CTT TCT AGC TTC TGG ATC ATC ATC GAT AG 3’

Supplemental Table 2. Primers used for cloning of UPF3B truncation constructs (above) and UPF3B mutations and Avi-tagged variants (below).
